# Exploring Flexibility of Progesterone Receptor Ligand Binding Domain Using Molecular Dynamics

**DOI:** 10.1371/journal.pone.0165824

**Published:** 2016-11-08

**Authors:** Liangzhen Zheng, Valerie Chunling Lin, Yuguang Mu

**Affiliations:** School of Biological Sciences, Nanyang Technological University, 60 Nanyang Drive, Singapore, 637551, Singapore; University of Akron, UNITED STATES

## Abstract

Progesterone receptor (PR), a member of nuclear receptor (NR) superfamily, plays a vital role for female reproductive tissue development, differentiation and maintenance. PR ligand, such as progesterone, induces conformation changes in PR ligand binding domain (LBD), thus mediates subsequent gene regulation cascades. PR LBD may adopt different conformations upon an agonist or an antagonist binding. These different conformations would trigger distinct transcription events. Therefore, the dynamics of PR LBD would be of general interest to biologists for a deep understanding of its structure-function relationship. However, no apo-form (non-ligand bound) of PR LBD model has been proposed either by experiments or computational methods so far. In this study, we explored the structural dynamics of PR LBD using molecular dynamics simulations and advanced sampling tools in both ligand-bound and the apo-forms. Resolved by the simulation study, helix 11, helix 12 and loop 895–908 (the loop between these two helices) are quite flexible in antagonistic conformation. Several residues, such as Arg899 and Glu723, could form salt-bridging interaction between helix 11 and helix 3, and are important for the PR LBD dynamics. And we also propose that helix 12 in apo-form PR LBD, not like other NR LBDs, such as human estrogen receptor α (ERα) LBD, may not adopt a totally extended conformation. With the aid of umbrella sampling and metadynamics simulations, several stable conformations of apo-form PR LBD have been sampled, which may work as critical structural models for further large scale virtual screening study to discover novel PR ligands for therapeutic application.

## Introduction

Progesterone receptor (PR) is a member of the nuclear receptor (NR) superfamily, which regulates a complex network of gene transcription [1]. PR together with progesterone, a steroid hormone, is important for female reproductive tissue development, differentiation and maintenance. Like other steroid hormone receptors, PR is composed mainly of an N terminal domain (NTD), a DNA binding domain (DBD) and a ligand binding domain (LBD) [2,3].

Human PR is encoded by the *PRG* gene, which uses two promoters to express different gene products, PR-B and PR-A. These two PR isoforms (PR-B and PR-A) are almost identical within their DBD and LBD sequences where there are two activation function (AF1 and AF2) regions. The major difference is that PR-A lacks the 164 amino acids that are presented in PR-B at its N-terminus [2,4,5]. The NTD in PR-B contains another activation function (AF3) which may enable PR-B to work as a much stronger transcription factor revealed in the cell experiment and mouse model [6,7]. Besides that, more differences between PR-A and PR-B exist in physical effects. For example, the antagonist RU486 acts as a pure antagonist upon binding PR-A; however, it is a partial antagonist of PR-B [2,8]. Furthermore, the two isoforms may activate different subsets of genes [9]. More recent studies showed that the two isoforms contribute differently to the progress of breast cancer [10], and the ratio of PR-A and PR-B could regulate the mammary gland stem cell population in mouse model [11]. However, the underlying mechanisms of the distinct roles of the two isoforms still remain unrevealed.

**Fig 1:**
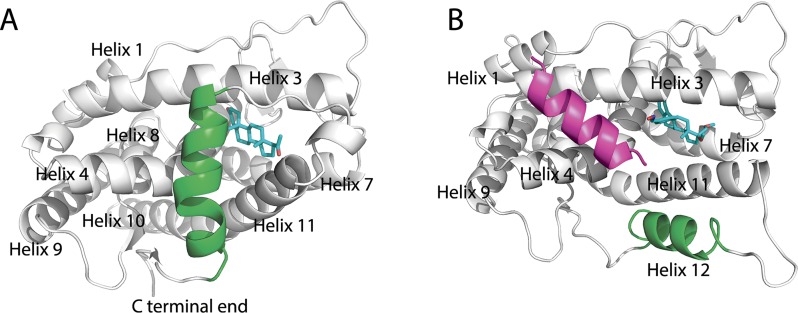
Crystal structure models of PR LBD. (A), holo-form agonistic conformation of PR LBD. (B), antagonistic conformation of PR LBD. Helix 12 and the co-peptide are green and magenta color, while for ligands (progesterone and asoprisnil), carbon atoms are cyan; oxygen atoms are red and nitrogen atoms are blue.

PR LBD, together with other NR LBDs, shares a globular three-layer folding style formed by 11 α-helices (helix 2 is missing in PR LBD) and 1 β-turns [2]. Generally, there are distinct conformation types of NR LBDs’ structures: the apo-form (non-ligand bound) conformation, holo-form conformation (with ligand bond), which again further could be divided into two types, the agonistic conformation and the antagonistic conformation. The extended apo-form conformation of NR LBDs was firstly described in retinoic X receptor-α (RXRα) [12], where helix 12 is totally dispatched from the surface of the LBD core and 12 does not have any hydrophobic interactions with the LBD. Similarly, the apo-form ERα also employs such an extended conformation [13].However, as far as we know, no apo-form PR LBD structural models have been deposited in RCSB Protein Data Bank (PDB). Structures of other holo-form NR LBDs have also been solved by crystallography. The comparison of these structures reveals that helix 12 in NR LBDs shows large flexibility while the other helices remain a similar architecture. The first holo-form agonistic PR LBD structure model (PDB ID 1A28, Fig 1A), which adopts a similar anti-parallel sandwiched helices folding pattern as proposed in other NR LBDs, was published in 1998 [14]. In this structural model, the LBD adopts the classic agonistic conformation with progesterone co-crystalized in the binding pocket. Several groups suggested that the Met909 in the N-terminal end of helix 12 is important for the stabilization of helix 12 in agonistic conformation. Firstly, the side-chain of Met909 is deeply buried and hooked by the hydrophobic interaction with the progesterone [15]. Besides, the side-chain of another residue Glu723 in helix 3 anchors the backbone of Met908 and Met909 by a so-called dipole system [14,16]. These interactions may play vital roles for fixture of the N-terminal part of helix 12 close to helix 3 to creates a “hydrophobic cleft” for LXXLL motif containing co-peptide of steroid receptor coactivators (SRCs) binding [3]. After homologous dimerization, PR is thus translocated into the nucleus and the dimer further binds to specific DNA sequences, progesterone response elements [17], through its DBDs, subsequently mediating the transcription activation [2,3,18].

The holo-form antagonistic conformation of PR LBD is characterized by the displacement of helix 12. Different from the agonistic conformation of NR LBDs, helix 12 is prevented from covering the ligand binding pocket, or mimicking the binding position of a co-activator, therefore prohibiting a co-activator binding but facilitating a co-repressor binding [2]. Taking the crystal antagonistic structure models (PDB ID 2OVH, Fig 1B) as an example, helix 12 is displaced from the surface of the LBD core and is antiparallel to helix 11, while the co-peptides (12 residues), which are a the common sequences in NCoR peptide (25 residues), occupy the hydrophobic groove formed by helix 3 and helix 4. The antagonists of PR LBD bind to the same binding pocket as progesterone does, whereas the unusually large 11β-substituted group of some antagonists prevent helix 12 from contacting with helix 3 [19,20]. In more detail, the antagonist 11β-substituted group adopts the conformational space where Met908 and Met909 in helix 12 used to take, therefore it was believed the relatively large size of 11β-substituted group contributes to the antagonistic conformation [19]. Interestingly, among the antagonists, the previously recognized antagonist RU486, was found to also be able to bind to PR LBD in a destabilized agonistic conformation [16]. However, helix 12 in this RU486-bound PR LBD complex has higher values of the b-factor, which indicates a more flexible nature of helix 12 in this conformation. Besides, two selective progesterone receptor modulators (SPRMs) Org3H and asoprisnil, also bind to PR LBD in its agonistic conformation [21]. The reduced antagonistic activity and increased agonistic activity of PR may result from the agonistic conformation formation upon Org3H binding. The fact that agonists (progesterone and progestin), full-antagonist (RU486) and SPRMs (asoprisnil and Org3H) all are able to bind to the agonistic conformational PR LBD, indicates that the binding pocket is flexible enough to accommodate ligands with different sizes and chemical species [2,22].

To our knowledge, no apo-form PR LBD structure model has been proposed, and no simulation study on the PR LBD has been carried out so far. In this study, we are the first to explore the conformational dynamics of PR LBD, especially apo-form PR LBD, using molecular dynamics simulation method and advanced sampling techniques (umbrella sampling and metadynamics). Several stable apo-form conformations of PR LBD have been observed. And some key residues, such as Gln719, Glu723 and Arg899 have been found to be important for the dynamics of helix 12, helix 11 and the loop between them (named as loop 895–908). Lys919 in helix 12 may interact with Glu723 in helix 3 to stabilize helix 12 in apo-form PR LBD. And we also suggest that helix 12 in apo-form PR LBD may not adopt the extended configuration as in other NR LBDs, such as RXRα LBD and ERα LBD. The deepened understanding of the dynamics of apo-form PR LBD therefore opens the gateway for large scale virtual screening to search for novel ligands.

## Results

### The antagonistic conformation is intrinsically flexible

**Fig 2:**
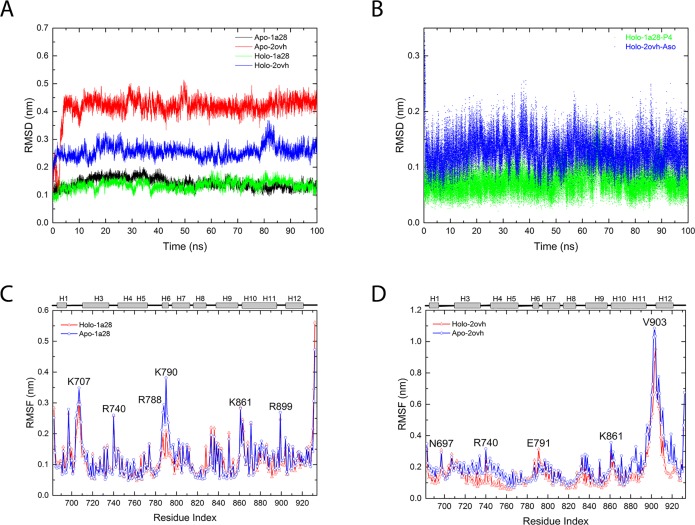
RMSDs and RMSFs data obtained from normal MD simulations. (A), The RMSDs of the first repeat trajectory for the 4 systems in Table 1, green and black for apo-1a28 and holo-1a28 simulations, red and blue for apo-2ovh and holo-2ovh simulations. (B), RMSDs of progesterone (P4) (green) and Asoprisnil (blue) in the PR LBD-ligand complexes. The RMSFs of the apo-1a28/holo-1a28 simulation (C) and apo-2ovh/holo-2ovh simulations (D), where residues’ index with rather large RMSF values are indicated. The secondary structures of LBD are displayed at top of panel C and D, whereas “H” indicates an α-helix.

During the normal MD simulations, αC atom RMSDs of the LBD go up quickly in the first 10 ns for both apo-2ovh and holo-2ovh simulation systems (described in method part), while in the agonistic states, RMSDs of both apo-1a28 and holo-1a28 systems keep lower than 0.2 nm (Fig 2A). Clearly the protein is more flexible in the antagonistic conformation than in agonistic conformation. Not only the LBD, agonist progesterone (in holo-1a28 system) has smaller RMSD comparing antagonist asoprisnil (in holo-2ovh system) (Fig 2B). Again, this suggests a less mobile instinct of agonistic conformation than antagonistic conformation in PR LBD. Consistently, the residue RMSFs of the agonist systems (apo-1a28 and holo-1a28) (Fig 2C), are smaller than those of the antagonist systems, apo-2ovh and holo-2ovh respectively (Fig 2D). Interestingly, the RMSFs of one residue Lys790 show large difference between apo-1a28 and holo-1a28 systems. Later analysis reveals that the different behaviors of Lys790 are important for the dynamics of two loops (loop785-808 and loop 703–712). Furthermore, the residue RMSFs of the C-terminal end of helix 11, loop 895–908 (the loop between helix 11 and helix 12) and helix 12, are relatively higher than other regions (Fig 2D) in apo-2ovh and holo-2ovh systems. Thus this helix-loop-helix segment of helix 11, loop895-908 and helix 12 is the most flexible regions in the antagonistic conformation of PR LBD.

**Fig 3:**
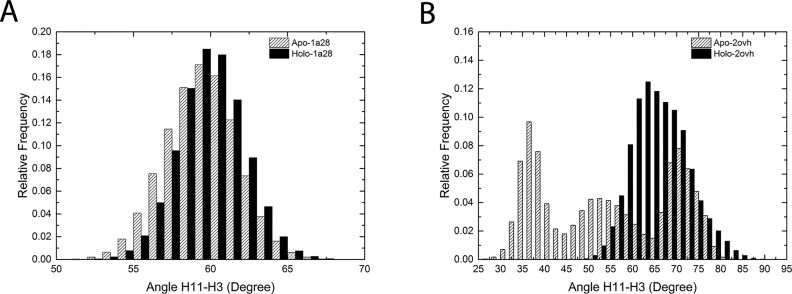
The frequency distribution of the crossing angle between helix 11 and helix 3. (A), the helix 11-helix 3 cross angle frequency distribution in apo-1a28 and holo-1a28 simulations. (B), the helix 11-helix 3 cross angle frequency distribution in apo-2ovh and holo-2ovh simulations.

The relative orientation of helix 11 with respective to helix 3 turns out to have different patterns in the 4 normal MD simulations. The distributions of the crossing angle between helix 11 and helix 3 show clear difference between the antagonistic states and the agonistic states: the distribution (about 15° angle range) is narrow for both apo-1a28 and holo-1a28 systems shown in Fig 3A; in apo-2ovh system, the crossing angle has three peaks spanning a 50° angle range, whereas in holo-2ovh system, the cross angle values are focused on a 30° range (Fig 3B). The large variation range of the crossing angle suggests the flexibility of helix 11 as well.

Overall, the PR LBD in antagonistic conformations is more flexible. This finding is consistent with the fact that, in the crystal structure models of the antagonistic conformations, the coordinates of the loop 895–908 between the helix 12 and helix 11 are missing, while the b-factors of the partial helix 11 and whole helix 12 are higher than other regions [19].

### Loop 785–808 and loop 703–712 are dynamically correlated with progesterone binding

**Fig 4:**
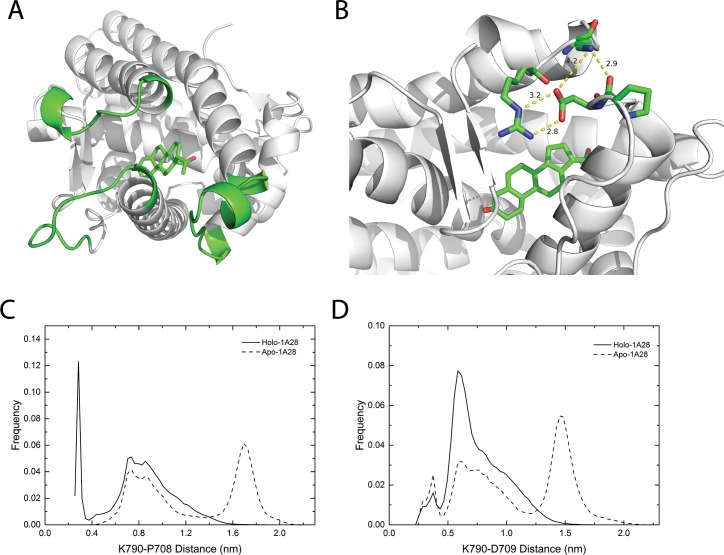
The interaction and distance relative distributions between residues in loop 786–808 and loop 703–712. (A), the overview of three important loops (green color) in holo-form crystal agonistic conformation (PDB ID 1A28). (B), the detailed view of the interactions between the residues in loop 703–712 and loop 786–808; the LBD structure is the last frame of a 100 ns trajectory of holo-1a28 simulation system; the yellow dashed lines indicate close contacts between atoms, the numeric labels are distances in unit of angstrom. (C), the relative distribution of the side-chain distance between Lys790 and Asp709 in apo-form and holo-form agonistic conformations. (D), the relative distribution of the distance between the Lys790 side-chain and the Pro708 backbone oxygen atom. In panel A and B, carbon atoms in the ligand progesterone are in green, oxygen atoms are in red color.

In our simulations, the agonist progesterone binding is found to change the dynamics of loop 785–808 and loop 703–712, which were reported to play vital role in ligand entry in PR LBDs [16,21,23] and ER LBDs [24]. Residues 785–800, especially Lys790, have smaller RMSFs in holo-1a28 system than apo-1a28 system (Fig 2C). One of the reasons could be that in holo-1a28 system, Lys790 interacts with the side-chain carboxyl group of Asp709 and the backbone oxygen atom of Pro708, both of which are located in another loop, loop 703–712 (Fig 4A and 4B). Lys790-Asp709 and Lys790-Pro708 distance distributions clearly demonstrate such interactions (Fig 4C and 4D): a sharp peak around 0.3 nm for Lys790-Pro708 and a peak around 0.7 nm for Lys790-Asp709 in holo-1a28 system, while for apo-1a28 system, the most populated distances between Lys790 and Asp709 and between Lys790-Pro708 are around 1.5 nm and 1.7 nm, respectively. Whereas, Arg788 also closely interacts with Asp709 and Asn709 (Fig 4B), however, the distance distributions of the Arg788-Asp709 and Arg788-Asn705 are quite similar in both apo-1a28 and holo-1a28 simulation systems (S1 Fig).

Though it has been reported that in ligand soak experiment, antagonist RU486 binding to agonistic PR LBD may alter the flexible loop 785–808 conformation [16], in our simulation, we noticed that in antagonistic LBD simulations (apo-2ovh and holo-2ovh systems), antagonist asoprisnil binding did not trigger the dynamics difference between loop 785–808 and loop 703–712 as in agonistic LBD simulations. Therefore it may indicate that ligand binding may be correlated with loop 785–808 and loop 703–712 dynamics for LBD in agonistic conformation, not in antagonistic conformation.

### Ligand binding alters globular dynamics of PR LBD

**Fig 5:**
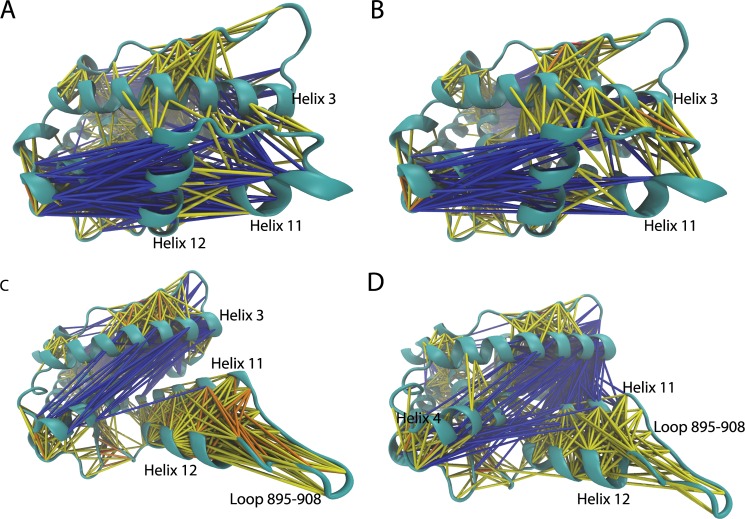
**The overall cross-correlation networks in simulation systems apo-1a28 (A), holo-1a28 (B), apo-2ovh (C) and holo-2ovh (D).** If two residues are correlated or anti-correlated, they are linked by lines. Different cross-correlation co-efficiency (ccc) is indicated by color. Anti-correlation is blue (-0.4 < = ccc<0.2), yellow (0.2< = ccc<0.4), orange (0.4< = ccc<0.6) and red (ccc> = 0.6) are used for correlations. The cross-correlation maps of these 4 systems are provided in S2 Fig.

Cross-correlation analysis of LBD residues provides a tool to decipher the hidden dynamical relations among different parts of a protein. Most parts of the LBD in the agonistic conformation have similar correlation patterns in both apo-form and holo-form. However, both loop 895–908 and C terminal part helix 11 have stronger correlations with helix 3 in holo-1a28 system (Fig 5A) than apo-1a28 system (Fig 5B). Interestingly, the anti-correlations are much stronger for helix 11 and helix 12 in apo-1a28 system. The reason of the enhanced correlations between loop 895–908 and helix 3 and between helix 11 and helix 3 in agonistic conformations comes from increased stability of loop 895–908 and helix 11, as well as helix 12 whose stability is critical to maintain the agonistic conformation. Ligand binding (and co-peptide binding) also changes the cross-correlation patterns in the antagonistic state. In apo-2ovh system, helix 12 and helix 11 have intensive correlations, which is partially lost in holo-2ovh system (Fig 5C). With ligand and co-peptide binding, the anti-correlation between helix 11 and helix 3 are significantly increased. Similarly, the anti-correlation between helix 4 and helix 12 are also much more intensive in holo-2ovh system than apo-2ovh system (Fig 5D).

It should be noted that normal MD simulations only sample rather local motions around the initial structures in a finite time scale. Thus different cross-correlation behaviors we observed may not be applicable to much longer time scale [25].

### Population distribution of apo-form PR LDB between agonistic and antagonistic conformations

**Fig 6:**
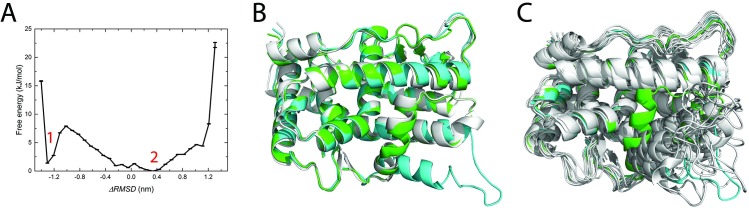
Free energy along the Δ*RMSD* coordination and the low free energies representative structures. (A), the free energy along the one dimensional coordination together with error bars. (B), the representative structure (gray color) in the local minimum 1, where Δ*RMSD* = -1.3 nm. (C), 10 representative intermediate structures in a local minimum 2 (the Δ*RMSD* range 0.3 nm to 0.5 nm). In panel B and C, the PR LBD crystal agonistic conformation (green) and the crystal antagonist conformation (cyan) are also superimposed with the representative structures.

Δ*RMSD*, the one-dimensional coordination, is employed in the umbrella sampling method. This Δ*RMSD*, which has been explained in method part, is defined as the difference of RMSDs with respect to the two reference crystal structural models (PDB ID 1A28 and 2OVH respectively). The more negative value the Δ*RMSD* is, the more close the structure is towards the agonistic conformation. The free energy as a function of the Δ*RMSD* constructed in the umbrella sampling indicates multiple intermediate states exist for the apo-form of PR LBD. There are two major local minima (labeled as umL1 and umL2) along the Δ*RMSD* (Fig 6A). The energy barrier between these two local minima is around 6.5 kJ/mol. Thus the transition between the two local minima might not be a rare event. For the umL1 where Δ*RMSD* = -1.3 nm, the frames within around -1.4 nm and -1.2 nm were extracted and clustered with a cutoff of 0.2 nm. Only one cluster was discovered. The cluster representative structure turns out to be quite close to the crystal agonistic conformation (Fig 6B). While for umL2 (Δ*RMSD* = 0.3 nm), the representative structures (labeled as uL2.1 to uL2.10) of the first 10 largest clusters of the frames with Δ*RMSD* between 0.2 nm and 0.4 nm are presented together with the reference structures. The structures from umL2 have diverse configurations in loop 895–908 and helix 12 (Fig 6C). For example, the representative structure (labeled as uL2.2) of the 2^nd^ largest cluster in umL2 shows that the N terminal part of loop 895–908 is close to helix 3 and Glu723 in helix 3 interacts with Arg899 in loop 895–908 to draw helix 12 near to helix 3. The interaction network around the helix-loop-helix segment of this uL2.3 structure model thus is quite similar to one of the apo-2ovh system normal MD simulation representative structures (S3 Fig), aL2 (S4 Fig). In other representative structures, however, loop 895–908 is pointed outward from the LBD surface and does not contact with other residues in PR LBD.

### Free energy surface of apo-form PR LBD

For apo-form PR LBD, there may not be a direct transition pathway from the antagonistic conformation towards the agonistic conformation, or vice versa, thus the one-dimensional path coordinate Δ*RMSD* may not depict a complete dynamic picture of the protein. Therefore, two other collective variables (CVs) have been chosen to sample the apo-form PR LBD conformations with the method of metadynamics simulation. These apo-form PR LBD models sampled from metadynamics would be valuable for further researches, such as large scale virtual screening against PR LBD.

**Fig 7:**
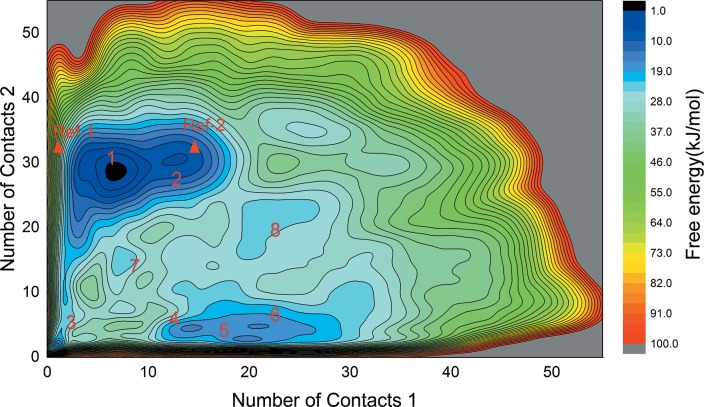
The free energy surface (FES) map constructed by metadynamics simulations. 8 major basins of local minima are labeled together with the positions of two crystal structure models (Ref-1: 2OVH; Ref-2: 1A28) indicated by red triangle marks. The color key at right side of the figure indicates the free energy scales in unit of kJ/mol. The iso-surface lines are given every 10 kJ/mol.

The free energy surface (FES) constructed based on the two NC CVs (NC1 and NC2, explained in method part) in our well-tempered metadynamics is presented in Fig 7. Eight major local minima have been labeled as L1 to L8, where L1 dominated as the largest local minimum in this FES map. From the plot, the crystal structure of the antagonist-bound conformation (at NC1 = 0.99 and NC2 = 34.08) is not in a low free energy basin for apo-form LBD, while the crystal structure of the agonist-bound conformation (at NC1 = 16.69 and NC2 = 31.24) is at the edge of L2. This further indicates that, as revealed in normal MD simulations, in apo-form PR LBD, the antagonistic conformation is less stable than agonistic conformation. However, crystal agonistic model is not the most stable apo-form conformation. The representative structures (S5 Fig) of these 8 local minima, labeled as mL1 to mL8, are extracted by clustering analysis. Except the flexible helix-loop-helix segment in PR LBD, the other regions could be well-superimposed with crystal agonistic and antagonistic models (S5A Fig). Similarly, we noticed that the representative structures (S3 Fig), sampled in normal MD simulations of apo-2ovh and holo-2ovh systems based on dPCA analysis, are quite flexible in this segment as well. Though, the relatively short time scale for normal MD simulations are not sufficient enough to sample all conformations in this helix-loop-helix segment, some common interactions were observed in both normal MD and metadynamics simulations.

For these representative structures sampled in metadynamics in the most flexible helix-loop-helix segment, some common interaction pairs have been summarized (Table 1). Glu723 in helix 3 interacts with Lys919 and Gln916 to stabilize the C terminal part of helix 12, thus helix 12 adopts a configuration with a quite flexible N terminus, with only one exception in representative structure mL3. Another pattern, the salt bridge interactions between Arg899 with Glu723 and Gln719 are also observed in the representative structures mL1, mL2 and mL3, where the adjacent Ser898 interacts with Gln916. These interactions were also observed in normal MD simulations (S4 Fig).

**Table 1:** Interaction pairs involving residues in the helix-loop-helix segment in metadynamics low energy structures.

Structures	Interaction pairs in helix 3, helix 12 and loop 895–908
L1	Asn719-Ser898,Glu723-Lys919, Glu723-Arg899
L2	Asn719-Ser898, Asn719-Arg899, Glu723-Arg899, Glu723-Lys919, Trp755-Pro918*
L3	Asn719-Ser898, Asn719-Arg899, Glu723-Arg899
L4	Glu723-Ser902, Glu723-Gln916, Glu723-Lys919
L5	Glu723-Gln916, Asn719-Ala900*
L6	Glu723-Gln916, Glu723-Lys919, Asn719-Ala900*, Arg899-Ala900*
L7	Glu723-Gln916, Glu723-Lys919, Trp755-Lys919*
L8	Glu723-Gln916, Glu723-Lys919, Arg899-Glu904, Ser898*-Thr716

* indicates the interacting atoms are on the backbone, whereas all other interaction pairs are on side-chains.

**Fig 8:**
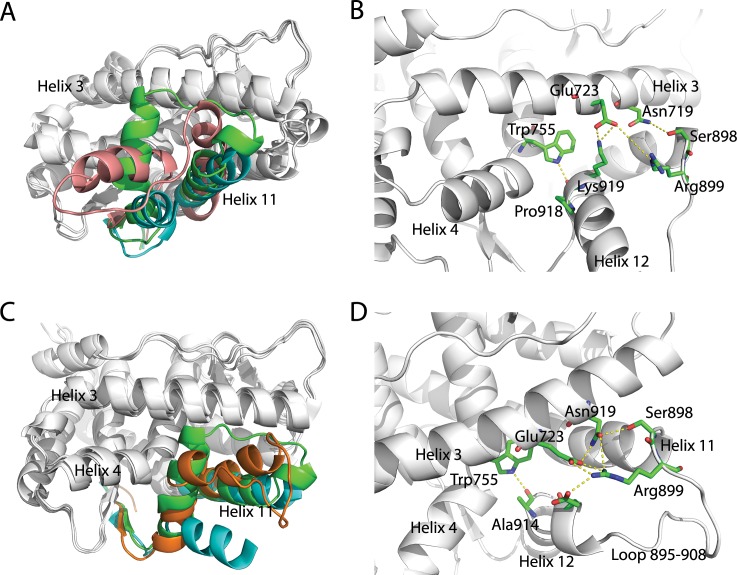
Representiave structures of local minima 1 and 2 sampled in the metadynamics simulation. (A), the representative structure mL1 (pink) superimposed with crystal agonistic and antagonistic models. (B), the detailed view of interaction networks around the helix 11, loop 895–908 and helix 12. (C), the representative structure mL2.3 superimposed with crystal agonistic and antagonistic models. (D), the detail interaction network between residues in helix-loop-helix segment. The crystal agonistic (PDB ID 1A28) and antagonistic (PDB ID 2OVH) conformations are colored as green and cyan in the helix-loop-helix region. The yellow dashed lines indicate close contacts between residue atoms.

Taken mL1 structure as an example, the overall architecture of PR LBD is quite distinct from the crystal agonistic or antagonistic conformations (Fig 8A and 8B). The N terminal part of helix 12 is pointed outwards and thus the binding pocket in LBD is directly exposed to solvent. The salt bridge interaction between Glu723 and Lys919 would lock the C terminus of helix 12, while residues in the N terminal of helix 12 are free of contacts, therefore contributing to the flexibility of the helix 12 N terminal part. And helix 11 is in a position more towards helix 3 than that in both agonistic and antagonist conformations. The cross angle between helix 3 and helix 11 in mL1 is around 75°, about 20° more than the cross angle in crystal agonistic and antagonistic models. The interaction pairs between Arg899-Glu723, Arg899-Asn719 and Ser898-Asn719, would pull helix 11 towards the center of helix 3, thus it may account for the larger cross angle in this mL1 structure. For loop 895–908, it is in a disordered state, where defined secondary structure is not formed. The majority residues in loop 895–908 do not form any contacts with other residues in LBD, except a possible hydrogen bond between Ser902 (in helix 12) and Gln916. We also found that, a representative structure of the third most populated cluster at L2, labeled as mL2.3 (Fig 8C and 8D), is somehow similar to the crystal agonistic conformation, with a αC RMSD around 0.3 nm. For this mL2.3 structure, the hydrogen bonding or salt bridge interactions between Trp755-Ala914, Glu911-Glu723, Glu911-Arg899, Glu723-Arg899 and Asn719-Arg899, hold helix 12 in a position comparable to the crystal agonistic conformation. Through the metadynamics simulation, we have sampled a structural model close to the crystal agonistic conformation, which is one of the stable conformations of PR LBD in its holo-form.

So far no experimental determined structure of apo-form PR LBD is available to compare with our predicted structural models directly. To verify our models, each representative structure of the local minima recruited as the initial structure for a short normal MD simulation. Most of the representative structures (except mL3) are stable during the 50 ns normal MD simulations (S6 Fig). These stable representative structures thus may describe the different states of apo-form PR LBD, and could be used for docking studies as different receptor models to search for potential ligands [26].

## Discussions

### Glu723 and Arg899 are important for the structural dynamics of PR LBD

The crystal structure of the agonistic conformation model suggests that Met909 [15] plays a key role in stabilizing of the helix 12 to adopt the position closely patched to the core LBD, and Glu723 interacts with the backbone of Met908 and Met909 to hook helix 12 [14–16]. Lusher et al [21] observed that PR LBD could remain its agonistic conformation with antagonist asoprisnil in the binding pocket. In their structure model (PDB ID 4A2J) [21], the asoprisnil 11β oxime group forms a hydrogen bond with the side-chain of Glu723 [14]. Another similar case is that the antagonist RU486 (mifepristone) binds to PR LBD also in the agonistic conformation [16]. Such antagonist-in-agonistic conformation has also been overserved in asoprisnil-LBD complex [21] and UPA-LBD complex [27]. These structure models highlight the importance of the interactions among Met908, Met909 and Glu723 for the maintenance of the agonistic conformation of PR LBD.

**Fig 9:**
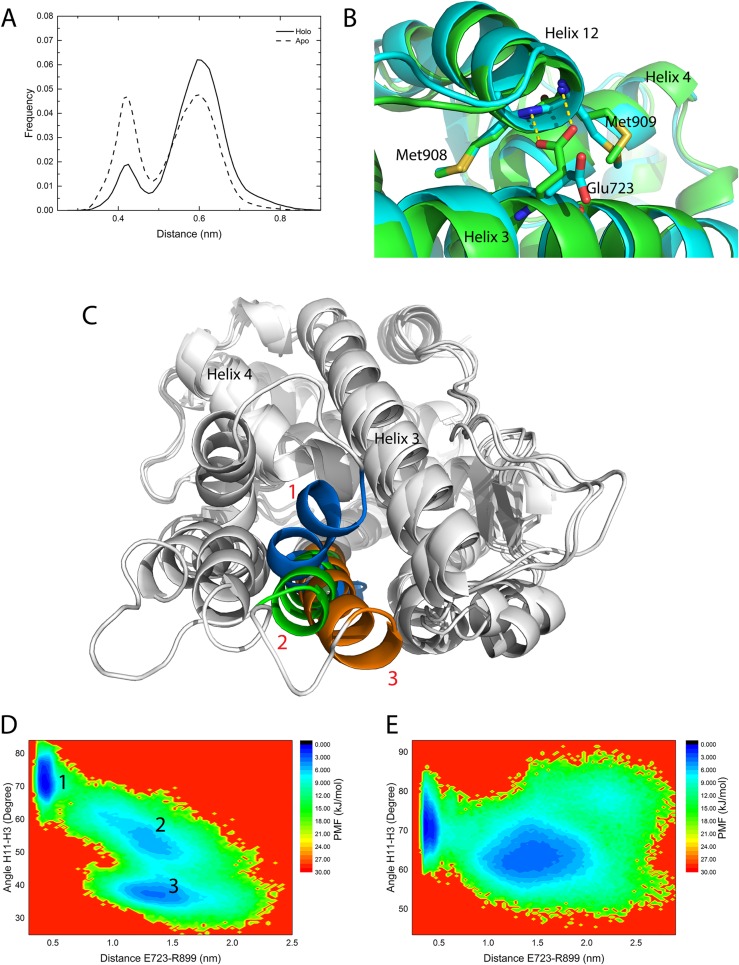
The interactions and distance distribution of Met908/Met909 and Glu723 and the cross angle between helix 11 and helix 3. (A), the frequency distribution of the backbone nitrogen atoms of Met908 and Met909 to Glu723 side-chain oxygen atoms distance. (B), the two distinct interaction patterns at the two distance frequency distribution peaks, if the distance between Met908, Met909 and Glu723 is 0.43 nm colored by green, if the distance is 0.6 nm, colored by cyan; the structures were extracted from clustering analysis based on the distance from the apo-form agonistic normal MD simulations. (C), the three states of helix 11 observed in apo-form antagonistic simulations. State 1 (marine) has a ~75° cross angle between helix 11 and helix 3, while the angles for state 2 (green) and state 3 (orange) are about 55° and 40°, respectively. State 1 and state 3 are from structural models of aL2 and aL3 (S3B Fig), while state 2 is the crystal antagonistic model (PDB ID 2OVH). (D and E), PMF maps of the helix 11-helix 3 cross angle in apo-2ovh and holo-2ovh simulations.

In apo-1a28 and holo-1a28 normal MD simulation systems, the distance between the Glu723 side-chain carboxyl group to the backbone nitrogen atoms of Met908 and Met909 is plotted as the frequency distribution (Fig 9A). The distance distribution of holo-1a28 system has one major peak, while for apo-1a28 system, two major peaks have similar heights which suggests that progesterone binding could shift the Glu723 from the on-state (with hydrogen bonding to Met908 and Met909) to the off-state (without hydrogen bond) (Fig 9B). Clearly the presence of the ligand weakens the Glu723-Met908/Met908 interactions. Arg899 is at the C terminal end of helix 11 in PR LBD. The interaction between Arg899 and Glu723/Asn719 brings loop 895–908 close to helix 3, and also changes the position of helix 11. For the position of helix 11, there exists mainly three states sampled from normal MD simulations as shown in Fig 9C, representing three different helix 11-helix 3 cross angles, around 75°, 55° and 35° respectively. The PMF as a function of two variables, one being the helix 11-helix 3 cross angle and the other being the distance between the side-chains of Glu723 and Arg899 is constructed (Fig 9D and 9E). A low energy basin stands out with a small Glu723-Arg899 distance (about 0.4nm) and large cross angle (around 75°). This lowest free energy basin indicates that, in both apo-2ovh and holo-2ovh normal MD simulations, when Glu723 forms salt bridges with Arg899, the cross angle between helix 11 and helix 3 is locked around 65–80° (Fig 3). Meanwhile, the C terminal end of helix 11 is shifted more towards the center of helix 3. While Glu723 is far away from Arg899, the cross angle between the two helices may fluctuate in a rather large range, and helix 11 may shift more outwards from the core LBD thus having a quite small cross angle (around 35°), or helix 11 could be stabilized around a 55° cross angle with helix 3, which are commonly seen in the crystal agonistic and antagonist conformational models. In general, helix 11 dynamics are related to the Arg899-Glu723 salt bridge interaction.

**Fig 10:**
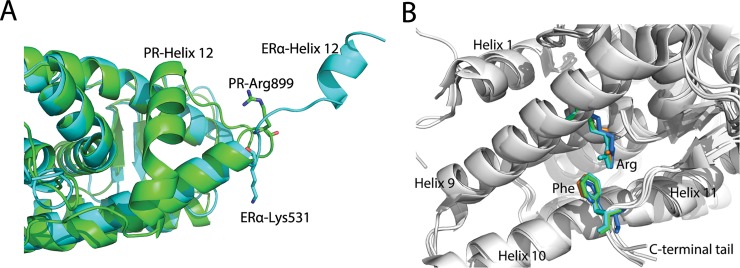
Overlay of apo-form ERα LBD with PR LBD and the π-cation interaction in NR LBDs. (A), ERα LBD (cyan, PDB ID 1A52) has a relatively shorter helix 12 and doesn’t has a extended C terminal tail, whereas PR LBD (green, PDB ID 1A28) has a longer helix 12 and a C terminal tail. (B), conserved arginine (in helix 9) and phenylalanine (in C terminal tail) are shown as sticks, whereas other parts are shown as cartoon. Green, PR LBD agonistic conformation (PDB ID 1A28); cyan, PR LBD antagonistic conformation (PDB ID 2OVH); marine, AR LBD agonistic conformation (PDB ID 4OEA); orange, GR LBD agonistic conformation (PDB ID 3K23).

One previous simulation study [28] proposed that in ERα LBD, the hydrogen bonding network, formed by His524-Glu399 and Glu419-Lys531, is a key factor for the stability of helix 11 and helix 3, further creating a “mouse trap” space for helix 12.[4,12,28,29] Interestingly, in PR LBD, Arg899 being in the similar position as Lys531 in ERα LBD could also hold helix 11 and helix 3, though in a different way (Fig 10A). Ser898 sometimes is also involved in the interaction with Asn719. Amino acid sequence alignment of several *Homo sapiens* NRs suggests that Glu723 is conserved in PR, AR, GR and mineralocorticoid receptor (MR), while Ser898 is conserved in PR, AR [14,30]. Further structural-based alignment revealed that the orientation of this serine is almost identical. Nevertheless, Arg899 is not as conserved as Glu723 and Ser898, while it is a lysine in GR, a histidine in AR and MR [30]. Interestingly, the fact that arginine, lysine and histidine all are positively charged residues suggests that this site could form favorable charge-charge interaction or salt bridge interaction with the conserved Glu723 to modulate the dynamics of the NR LBDs.

### Helix 12 is not likely to adopt a totally extended conformation in PR LBD

In the crystal structure models of apo-form ERα LBD [13] and RXR-α [12], helix 12 adopts an extended conformation and is totally dispatched from the core LBD thus exposes the ligand binding pocket to solvent. According to these crystal structure models, helix 12 must undergo large conformational changes to form the agonistic or antagonistic conformations. However, several studies cast doubts on this extended-helix 12 model in NR LBD [30–32].

The fast transition (within 5 ns) of the helix 12 from the extended conformation to an intermediate stable conformation in apo-form ERα LBD has been captured in a MD simulation study [28]. The fast change of the apo-form indicates that helix 12 may adopt several conformations regardless with or without ligand or co-peptide binding. Batista et al [31] modeled the time-resolved fluorescence anisotropy decays to explore the flexibility of the holo-form PPARγ LBD by attaching a fluorescent probe to the C terminal tail of the PPARγ LBD. They tested the model of ERα LBD and RXR LBD as well. In their work, only a relatively local motion of helix 12 in different NR LBDs had been observed. In the same time, we also performed two long normal MD simulations (over 1000 ns) of apo-form PR LBD started from the two crystal agonistic and antagonistic conformations. It turns out that helix 12 only has rather local motions resemble the stable states recovered in the short normal MD simulations. Besides, the extended helix 12 of PR LBD was never samples in all the simulations, thus it further indicates that, unlike ERα LBD (Fig 10A), the extended conformational PR LBD is a rare event at least in molecular dynamics simulations.

The shorter helix 12 (about 8 residues) in ERα LBD may be stable in the extended conformation. Other NRs with longer helix 12 and an extra C-terminal tail (12 residues in PR LBD) in LBDs, such as PR, AR and GR, the extended conformation (helix 12 totally dispatched from LBD surface) of LBD, has not been observed. This C-terminal tail is suggested to be involved in hormone binding and antagonist-dependent repressive function in PR, AR and GR [14,33–36]. In PR LBD, the C terminal tail forms a short antiparallel β-sheet, similar to GR LBD and AR LBD. Besides, in both X-ray agonistic and antagonistic models of PR LBD, the π-cation interaction between Arg845 and Phe930 were observed (Fig 10B). During our normal MD simulations, Arg845 and Phe930 are maintained in close contacts in different simulation systems (S7 Fig). What’s more, it turns out that these two highly conserved residues have counterparts, which could also form a π-cation interaction in other NR, such as AR and GR [14] (Fig 10B). The favorable π-cation interaction between a phenylalanine and an arginine may contribute a -2.9 ± 1.4 kcal/mol binding energy [37], therefore this Phe943-Arg845 pair further stabilizes the C terminal tail dynamics, which would subsequently restrain the helix 12 flexibility. Thus we propose that in apo-form PR LBD, helix 12 would not adopt a totally extended conformation as in apo-form ERα LBD.

### Simulating apo-form PR LBD targeting novel inhibitor discovery

From normal MD simulations, water molecules were found moving around the edge of the agonist or antagonist binding pocket in apo-1a28 and apo-2ovh simulations. These observations indicate a rather hydrophobic binding pocket as already been previously reported, which explains why known ligands for PR LBD are mainly steroid derivatives or other hydrophobic molecules [2,14,19,38–40]. This information suggests that a more focused ligand collection may be beneficial for virtual screening in this binding pocket.

In this study apo-form MD simulations were initiated from holo-form structures. Such approaches have been applied in HIV protease, where new allosteric pockets were detected [41]. In another study by Miao et al [42], simulations of apo-form M2 Muscarinic Receptor led to the discovery of several allosteric druggable sites or pockets. The newly detected sites or pockets are useful for future novel selective modulator designing. Similarly, we also detected four major druggable pockets (S8A Fig) based on apo-form simulations. The largest pocket (surrounded by green colored residues) has the pocket size ranging from 300 to 1400 Å^3^, consistent with the holo-form crystal models of the agonist and antagonist binding site [2], while the other three are close to the largest pocket however with small volumes. This largest pocket (S8B Fig) we found is a coalescence of the agonist and antagonist binding site with the adjacent pocket between helix 3 and N terminal part of LBD. During the normal MD simulations without a ligand in the binding pocket, side-chains around the pocket are freely rotatable. Thus the smaller binding pocket can form. Clearly the binding pocket in PR LBD could be fitted by a variety of small molecules which may be dramatically different from the known ligands. Previous screening studies [43,44] against PR LBD were mostly based on the crystal holo-conformation. Making use of the new binding pockets would possibly lead us to the discovery of new category of druggable molecules.

## Conclusion

PR LBD is an important drug target and the exploration of the structural dynamics may provide valuable information for further virtual screening. In this study, we aimed at describing the dynamics of LBD in both apo-form and holo-form, and we are more interested in discovering the diverse apo-form conformations of PR LBD. The computational simulations proved the PR LBD antagonistic conformations are not stable and may undergo large structural changes in helix 12, helix 11 and loop 895–908. Further examining the different low free energy conformations revealed that several residues, such as Gln719, Glu723, Ser898 and Arg899, play vital roles in the dynamics of the helix-loop-helix segment (helix 11, helix12 and loop 895–908). Metadynamics simulation discovered that another group of residues, Gln916 and Lys919 in helix 12 could interact with Glu723 to stabilize the N terminal end of helix 12 in LBD’s apo-form. Meanwhile, several stable apo-form LBD conformations have been sampled and their relative free energies were measured. Those stable apo-form PR LBD conformations as a demonstration of the receptor flexibility would facilitate the future virtual screening process.

## Material and Methods

### Normal Molecular Dynamics Simulations

Normal molecular dynamics [45] simulation is a well-developed method for studying the dynamics of proteins and nucleic acids. In this study, we applied normal MD simulations to understand the dynamics of PR LBD with or without ligands (and the co-peptide). The normal MD simulations were performed with Gromacs packages [46]. The force field for PR LBD (and the co-peptide) was Amber99SB-ildn.[47] For ligands (progesterone and asoprisnil), the atomic charges were calculated with HF/6-31G* basis set using Gaussian09 package [48] following by restrained electrostatic potential (RESP) charge fitting using Amber11 [49] antechamber module, and tleap module was also used to build the topology for the ligands, while the bonding parameters were adopted from the amber gaff force field [50].

The initial structures were the agonistic and antagonistic conformation. Unless stated, the agonistic and antagonistic conformations in this research specifically refer to the crystal models, 1A28 (chain A) and 2OVH, respectively. Four simulation systems were constructed with 3 repeats for each system (Table 2). In simulation system holo-2ovh, PR LBD binds to the ligand asoprisnil and a co-peptide (12 residues, a part of a co-repressor SMRT). While the water molecules in the original PDB files were removed, the missing heavy atoms were added by DeepView [51], and missing residues in 2OVH structure model were modelled by Swiss Modeller online server (http://swissmodel.expasy.org/). All the systems were simulated in explicit TIP3P [52] water molecules (around 10,000 to 14,000). The distances between the edge of the water box and the protein surfaces were set as 1.0 nm. Sodium and chlorine ions were added to neutralize the systems and to maintain the salt concentration at biological relevant concentration, 0.15M. The systems firstly went through a 1000-steps energy minimization carried out by the deepest descent algorithm, following by an Berendsen barostat [53] equilibration in NPT ensemble for 1 ns at the pressure of 1atm and the temperature of 300 K, by restricting the positions of the solute heavy atoms with a constrain constant of 1000 kJ/(mol∙nm^2^). Then the product runs were performed in the NVT ensemble at 300 K. A 2 fs simulation time step was employed, while the temperature was updated by velocity rescaling thermostat [54] every 0.1 ps. Long range electrostatics were handled by the PME scheme [55] with a real space cutoff as 1.0 nm. And a 1.2 nm cut-off for van der Waals interactions was adopted. All the hydrogen atoms were fixed according to SHAKE algorithm [56], while all the covalent bonds between heavy atoms were constrained by applying the LINCS algorithm [57]. Repeat runs, where necessary, were carried out by assigning a random initial velocity for a same simulation system. Snapshots were stored in the trajectory files every 500 steps (1 ps).

**Table 2:** The setup of the 4 systems in normal MD simulations of PR LBD.

S/N	System Name	Initial structure	Ligand	Co-peptide sequence	Repeats	Time
1	Apo-1a28	1A28	None	None	3	100 ns
2	Holo-1a28	1A28	Progesterone	None	3	100 ns
3	Apo-2ovh	2OVH	None	None	3	100 ns
4	Holo-2ovh	2OVH	Asoprisnil	GLEAIIRKALMGK	3	100 ns

The 80 ns trajectories of the four simulation systems in Table 2 were collected and the 3 repeat run trajectories for each simulation system were merged to make a 240 ns trajectory for root mean square deviation (RMSD), root mean square fluctuation (RMSF), distance-frequency, dihedral PCA analysis (dPCA) and clustering analysis. The dPCA analysis were performed according to the method by YG Mu et al [58]. Representative structures of low energy basins (local minima) were discovered based on clustering analysis. Basically, we first extracted all the frames (from MD trajectories) located in a low energy basin, and then performed the clustering analysis to discover the most populated clusters, where the center structure, whose distance to other members in the same cluster is minimized, was further extracted and defined as the representative structure of the low energy basin. Clustering analysis was carried out using Gromacs g_cluster utility tool and gromos clustering algorithm [59] with a 0.2 nm cutoff on the αC atom of residues in PR LBD. Side-chain distances between residues were calculated by Gromacs g_dist tool measuring the distance between their function groups (on the side-chains). For example, the distance between an arginine and a glutamic acid is the distance between the center of the three nitrogen atoms of the arginine side-chain and the center of the two oxygen atoms in the glutamic acid side-chain. If the side-chain distance between two residues is less than 5 ‎Å, then these two residues are in close contact. π-cation interaction [60] is defined when the distance between the centroid of an aromatic ring and the positive charged center is less than 6 ‎Å, also the angle between the line pointing from the aromatic ring center to the cation center and the aromatic ring plane normal is within 60° to 120°. The computation of the crossing angle between two α-helices was performed with Plumed 2.1 [61]. The method is to compute the two vectors along the two α-helices. The vector of an α-helix is defined as such that the vector points from the center of mass of the α-helix N terminal residues to the center of mass of the same α-helix C terminal residues. In our study, we chose the center of mass (COM) [62] of αC atoms from residue index 883–886 as the start point of the vector representing helix 11, the COM of αC atoms of residues 894–897 as the end point. Similarly, residues 712–715 and residues 730–733 were chosen for the starting and ending points of the vector for helix 3. PMF analysis [63], or free energy profile, was performed based on the probability distribution of reaction coordinates. The PMF is defined by probability of information transfer as following: $PMF=-k_{B}T\;\ln\frac{P_{i}}{P_{max}}$, where *k*_*B*_ is Boltzmann constant, *T* is the temperature (300 K in all the simulations), while *P*_*i*_ is the probability in the specific bin along reaction coordinates and *P*_*max*_ is the largest probability among all the *P*_*i*_ values. By this PMF calculation, we could estimate the relative free energy as a function of that reaction coordinate. The cross-correlation and residue interaction network analysis was performed by Wordom 0.23 [64,65]. The Pearson cross-correlation coefficiency (ccc) *C*_*ij*_ is defined in the following equation:

$C_{ij}=\frac{\langle {\Delta \vec{r}}_{i}∙\Delta {\vec{r}}_{j}\rangle }{\sqrt{\langle {\Delta {\vec{r}}_{i}}^{2}\rangle \langle {\Delta {\vec{r}}_{j}}^{2}\rangle }}$, where ${\Delta \vec{r}}_{i}$ is the displacement of residue *i* to its mean position. If *C*_*ij*_ = 1, it means residue *i* and *j* are fully correlated; while *C*_*ij*_ = -1, then it suggests that residue *i* and *j* are anti-correlated in motion. Based on the residue correlation map, by Visual Molecular Dynamics (VMD) package [66,67], we built the correlation network to distinguish the correlations between residues in different conditions. If residue index *i* and *j* are within 10 (|*i*-*j*| < = 10), the correlations between residue *i* and j are ignored. This step is applied to remove the correlations due to the special closeness. In this study, we computed the correlation coefficient from the last 40 ns normal MD simulations of each of the 12 trajectories. In each trajectory, we calculated the correlation in 10 ns blocks and used the average of correlation matrices as the final correlation. Only the αC atoms of the residues were considered for correlation analysis. For the same simulation system, the correlation matrices were the averaged correlation matrix from the repeat trajectories. VMD 1.9 was used to visualize the interaction networks. Pocket detection and pocket size calculation were performed with Fpocket 2 [68], where default parameters were adopted.

### Umbrella Sampling

Umbrella sampling method is an efficient tool which could be applied to explore the transition between two states of a macromolecule [69–71]. In a previous study by Wang et al [71], they combined NMA with umbrella sampling to study the large scale conformational changes in ligand-bound and ligand-free states. For PR LBD, only the holo-form crystal structures of LBD are available, and no any crystal structure model for the apo-form PR LBD could be employed for the open-to-close transition pathway population shift analysis [71]. On the other hand, we have two very different conformations, the agonistic conformation and the antagonistic conformation of LBD, available. Thus it is interesting to explore the population distribution between the two conformations in ligand-free states.

For each window in the umbrella sampling, a harmonic bias potential *E*_*umbrella*_ = *k* ∙ (Δ*RMSD* − *C*_*i*_)^2^, where *k* is the force constant and *C*_*i*_ is the *i*th reference point along one dimensional Δ*RMSD*, was added on the Δ*RMSD* to restrict the structure not too far away from the transition path. The Δ*RMSD*, which was proved to be an efficient tool to explore possible state transitions [69–71], is the difference between the RMSD values referring to two distinct states, which are described in the following context. For the windows in the umbrella sampling, the initial structures were prepared so that the Δ*RMSD* in the successive windows has equal distance.

By incorporating the normal mode analysis [71], the imorph tool in iMOD software [72] could generate a possible structure transition pathway between the two given end points. Default setup was used for iMod imorph tool using NMA algorithm to generate a transitional trajectory from antagonistic to agonistic conformation. Then, 28 structures chosen together with the 2 initial made 30 windows in this umbrella sampling. And the difference of reference Δ*RMSD* of the successive windows (*C*_*i+*1_ –*C*_*i*_) was 0.1 nm.

In the umbrella sampling simulation, 30 parallel runs were carried out by Gromacs package [46] patched with Plumed 2.1 [61]. In detail, Δ*RMSD* is defined as Δ*RMSD* = *RMSD1* –*RMSD2*. For every single run in each window, the *RMSD1* and *RMSD2* were calculated every 1 ps by the optimal algorithm in Plumed 2.1. In each window, the instantaneous conformation in the simulation trajectory firstly was superimposed to the crystal antagonistic conformation structure on the backbone of the residue 683–902, and the αC atoms RMSD of residue 903–932 of PR LBD was calculated and used for *RMSD1*. The purpose of aligning on the residue 683–902 and calculating RMSD on residue 903–902 is to maximize the RMSD difference of the most flexible helix-loop-helix segment (helix 11, helix 12 and loop 895–908). Similarly, for *RMSD2*, the reference structure was the crystal agonistic conformation. The umbrella sampling procedure in our study was modified from the studies by Arora et al [69] and Wang et al [71]. For each window, the system firstly went through a 1000 step energy minimization with a 1000 kJ/(mol∙nm^2^) constraint on the heavy atoms. In the following 1 ns NPT equilibrium at 1 atm and 300 K, the constraint force constant on the heavy atom was decreased gradually from 1000 to 10 kJ/(mol∙nm^2^). For the product runs, a harmonic bias potential *E* (force constant *k* = 10 kJ/(mol∙nm^2^)) was applied on the Δ*RMSD*. The force constant for the bias potential was optimized in this study to ensure that the secondary structure of the LBD in each window would not be distorted. All other parameters, such as the force field and simulation conditions, were the same as in the normal MD runs. The product runs lasted 2 ns, since longer trajectories could not significantly change the results. The last 1.5 ns out of the 2 ns trajectories of each window was collected for the following WHAM analysis [73] to obtain the free-energy profile (or PMF), alone the Δ*RMSD* coordination. Errors of the free energy profile was estimated based on bootstrap method in WHAM analysis, though the error bars are quite small. The representative structures of the low energy minimum were extracted based on clustering analysis, which has been discussed in the normal MD method part.

### Metadynamics simulation

Metadynamics simulation [74–79] is a rather new method which enables us to explore the free energy surface based on chosen coordinations, or CVs, in a computation affordable time scale. Different from normal MD simulations, metadynamics is an enhanced sampling method. By adding bias potential alone the predefined CVs, metadynamics sampling could reconstruct the free energy surface (FES) based on the biased CVs and be able to accelerate the sampling process to capture rare dynamics events, such as the large scale conformation changes.

Identification of the stable structure models of apo-form PR LBD is of great interest to us. To achieve this goal, we performed well-tempered metadynamics simulation to construct the free energy surface (FES) based on two CVs, which have been detailed described in following text. Metadynamics is proved to be a useful tool to explore the FES of peptides folding [80,81], mutations in proteins [82,83], protein oligomerization [84] and complicate protein-ligand complex [85–87], and even has been implemented for enzyme catabolism. The efficiency of the metadynamics simulations is largely dependent on the choice of the CVs. The general CVs, such as distances and angles, in some circumstances, may not be enough to account for the complexity of our systems. For large protein or domains, the “good” CVs should be able to have certain degree of complexity. Some frequently used CVs for protein folding or large scale motions, for example, could be the number of contacts (NC) of αC atoms, the contact map, the hydrogen bonding number, anti-β RMSD [88], the α-helix RMSD [89], and also the path CVs [74]. Especially, the path CVs, which enable the system to explore a high dimensional transition path, are proved to be really powerful parameters to sample the transition pathway in many different systems [74, 85,86,89].

In our well-tempered metadynamics simulation [90,91], several CVs were defined. CV1, the number of αC atom contacts (NC1) between helix 12 (residue 908–922) and helix 3 (residue 713–734), and CV2, the number of αC atom contacts (NC2) between helix 12 (residue 908–922) and helix 11 (residue 882–898), were chosen for metadynamics biasing scheme. In order to obtain a smooth changing of the NC1 and NC2, a transformation equation was employed to calculate the value of NCs: ${NC}_{CA}=\sum_{i=1}^{N_{1}}\sum_{j=1}^{N_{2}}\frac{1-\frac{r_{ij}^{8}}{r_{0}^{8}}}{1-\frac{r_{ij}^{12}}{r_{0}^{12}}}$, where *r*_0_ is the distance cutoff for a contact. In this study, *r*_0_ = 0.85 nm, while *N*_*1*_ and *N*_*2*_ were the number of the αC atoms in the two respective helices. In addition, a wall bias was added on CV3, the α-helix RMSD [89] of the helix 12 (residue 908–922). The α-helix RMSD is a measure of the secondary structure distance to idealized α-helix in polypeptides. This CV3 was further defined by the following equation: $s_{\alpha }=\sum_{i=1}^{N}\frac{1-\frac{r_{i}^{8}}{r_{0}^{8}}}{1-\frac{r_{i}^{12}}{r_{0}^{12}}}$, where = 0.08 nm and is the RMSD distance between a standard α-helix. The wall bias could restrict the CV3 within the given region to avoid a totally deformation of the helix 12 thus facilitating the simulation to converge in a reasonable time scale. When the value of this CV3 is lower or higher than the cutoff value (5 and 10 in this study), a square-well bias potential was added to the system on CV3 according to the following scheme: $E_{wall}=\sum_{i}^{N}k_{i}{\left(\left(x_{i}-a_{i}+o_{i}\right)/s_{i}\right)}_{i}^{e}$, where the force constant = 300 kJ/(mol∙nm^2^), the rescaling factor = 1, the exponential factor = 2, the offset term = 0. Only if the instantaneous value of CV3, *x*_*i*_, is larger than the upper wall = 10 or smaller than the lower wall = 5, the bias potential is calculated and applied to the simulation system. In this equation, *i* is the number of the wall-potential biased CVs, here in this study, *i* = 1. In the simulation, the apo-form antagonistic conformation was used as the initial structure. The system setup, energy minimization, NPT equilibration and the setup for product run were employed similar to those in the normal MD simulations. The Gaussian width of NC1 and NC2 was chosen both as 1.0. This Gaussian width was chosen thus we could balance the simulation time and accuracy. And the height of the Gaussian potentials for NC1 and NC2, set as 0.2 kJ/mol, was deposited every 1 ps. The bias factor, which could define the dimension of the temperature in well-tempered metadynamics [91], was chosen as 10.

Approaching the end stage of the simulation, the convergence of the metadynamics was verified to ensure the simulation exploring the CVs’ space sufficiently. The FES maps, constructed based on NC1 and NC2 along the metadynamics simulation, were presented and towards the last 200 ns of the simulation, the FES map did not change much (S9 Fig). Besides, the free energy difference (Delta FES) between two local minimum along one CV space (NC1 or NC2) was plotted against simulation time, according to the method provided in Plumed 2.1 website (http://plumed.github.io/doc-v2.2/user-doc/html/belfast-6.html). For NC1, the free energy difference is the energy difference between two local minimum (NC1 values 5~9 and 12~15, respectively), while for NC2 the free energy difference is calculated based on another two local minimum (NC2 values 0~3 and 27~30). During the last 200 ns of the metadynamics simulation, the Delta FES curves for both NC1 and NC2 go through very small changes (within 1 kJ/mol) (S10 Fig). Therefore, we may conclude that the metadynamics simulation has reached the convergence criterion.

The frames located in low energy basins (local minima) in the final FES map were extracted and merged for further clustering analysis. The representative structure of the largest populated cluster in each local minimum was further recruited as the initial structure for a 50 ns normal MD simulation to verify the stability of these structures. Besides, these representative conformations, together with those sampled in umbrella sampling, are available upon requests. The apo-form conformations, sampled from metadynamics together with those from normal MD and umbrella sampling, were uploaded to FTMap webserver (http://ftmap.bu.edu/home.php) [92] for analysis and returned druggable scores per residue were collected. Those residues with high scores in different apo-form conformations were identified and grouped to predict druggable sites.

## Supporting Information

S1 Fig **Relative distance distributions for Arg788-Asn705 (A) and Arg788-Asp709 (B) side-chains.** (A), the distance between Arg788 side-chain amide group and the carboxyl group of Asn705 side-chain for apo-1a28 normal MD simulation and holo-1a28 simulation. (B), the distance between Arg788 side-chain amide group and the carboxyl group of Asp709 side-chain for apo-1a28 simulation and holo-1a28 simulation. Black line and dashed black line are used for apo-1a28 and holo-1a28 simulation systems respectively.
(PDF)

S2 Fig Cross-correlation coefficient (ccc) of αC atoms in normal MD simulation. The ccc maps of in apo-form (lower triangle) and holo-form (upper triangle) in agonistic (A) and antagonistic (B) normal MD simulations. The correlation matrices were constructed based on the method of normal MD simulation analysis. The secondary structures are displayed at top of the panels, where “H” represents an α-helix. The color scale is spanning from blue (ccc = -1, fully anti-correlation) to red (ccc = 1, fully correlation), whereas white (ccc = 0) stands for no correlation.
(PDF)

S3 Fig PMF maps of the first 2 eigenvectors in dPCA analysis and the representative structures in their local minima. (A), the PMF of the dPCA of the helix 11-loop-helix region in apo-2ovh simulations, where 7 local minima are identified and labeled. (B), the representative structures (labeled as aL1 to aL7) of each minimum in panel A. (C), the PMF of the dPCA of the helix 11-loop-helix 12 region in holo-2ovh simulations, where 9 major local minima are identified and labeled. (D), the representative structures (labeled as hL1 to hL9) of the 9 minima in panel C. From local minima 1 to 7 (panel B), or 1 to 9 (panel D), the representative structures are colored as green, sky blue, yellow, pink, magenta, cyan, orange, green cyan, blue, while the “Ref” labeled structure (gray color) is the crystal antagonistic conformation (PDB ID 2OVH) for reference.
(PDF)

S4 Fig Interactions between Arg899 and Glu723 in aL2 structural model (S3B Fig). Carbon atoms, nitrogen atoms and oxygen atoms are shown as green, blue and red respectively. Yellow dashed lines indicate close contacts between atoms.
(PDF)

S5 Fig The representative structures of the 8 major local minima in metadynamics. (A), superimposed representative structures. (B-H) detail interactions in flexible helix-loop-helix segment of representative structures from mL2 to mL8. Only the region (residues 892–922) is colored; other parts are shown in gray. Important residues in structures mL2 to mL8, shown as sticks, are colored as green, sky blue, yellow, pink, magenta, cyan, orange, and green cyan respectively.
(PDF)

S6 Fig RMSDs of the normal MD simulations of the representative structures of the local minima in metadynamics. During the 50 ns normal MD simulations, most of the representative structures stay stable (with αC atoms RMSDs are around or within 0.3 nm), while structure mL3 has a rather large RMSD fluctuations which indicates that this mL3 structure is not a real stable states.
(PDF)

S7 Fig Relative frequency distributions of side-chain distance between Arg845 and Phe930. The distance is defined between the COM of three nitrogen atoms in Arg845 side-chain and the COM of aromatic ring in Phe930. Black, red, green and blue lines represent the relative frequency of the distance in apo-1a28, apo-2ovh, holo-1a28 and holo-2ovh normal MD simulations respectively.
(PDF)

S8 Fig Druggable sites in PR LBD detected and binding pocket size. (A), the four druggable sites detected based on the representative conformations from normal MD, umbrella sampling, and metadynamics and plotted on crystal agonistic conformation; their outside residues are shown in different color, whereas the major binding pocket is surrounded green residues. And the other three druggable sites are formed by cyan, orange and magenta residues, respectively. (B) binding pocket size relative frequency distributions in normal MD simulations; black, red, green and blue lines represent the relative frequency of the binding pocket size in apo-1a28, apo-2ovh, holo-1a28 and holo-2ovh normal MD simulations respectively.
(PDF)

S9 Fig Free energy surfaces (FESs) constructed in different time windows during the metadynamics simulation. The FESs are constructed based on the Gaussians added from start of the simulation to a specific time point, such as 200 ns (A), 300 ns (B), 400 ns (C), 500 ns (D), 600 ns (E), 700 ns (F), 800 ns (G) and 900 ns (H). The color scales given at the right side of the figures indicate the free energy levels in unit of kJ/mol, while the iso-lines are drawn every 10 kJ/mol. The FESs for 0~700 ns (F), 0~800 ns (G) and 0~900 ns (H) are quite similar from a globular view, thus they could be an indication for the convergence of the metadynamics simulation.
(PDF)

S10 Fig The delta FES changes of one dimensional CV space along simulation progress. The delta FES changes of NC1 (CV1) and NC2 (CV2) during the whole simulation are presented by the red line and the blue line, respectively. Towards the large part (200 ns) of the simulation, the delta FES tends to be stabilized within a rather small range (1 kJ/mol), therefore it indicates the convergence of the metadynamics simulation.
(PDF)
