# Supplementary figures and images for: Exploring Flexibility of Progesterone Receptor Ligand Binding Domain Using Molecular Dynamics
Source: PLoS One. 2016 Nov 8;11(11):e0165824. doi: 10.1371/journal.pone.0165824 (PMC5100906; doi:10.1371/journal.pone.0165824)

**A**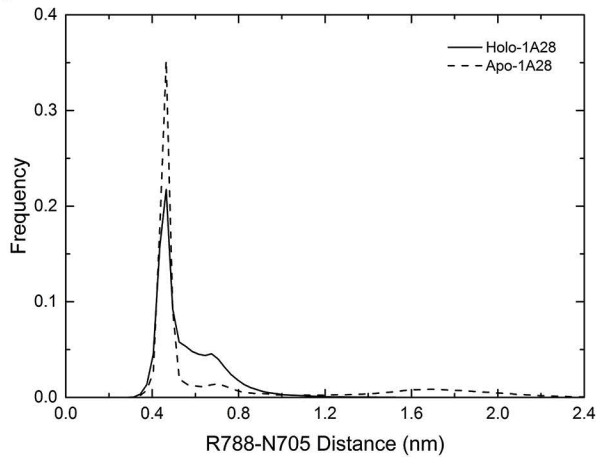**B**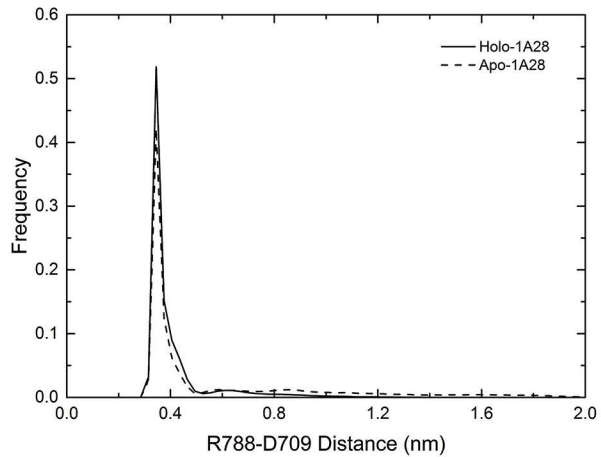

Supplement: S1 Fig — Relative distance distributions for Arg788-Asn705 (A) and Arg788-Asp709 (B) side-chains. (A), the distance between Arg788 side-chain amide group and the carboxyl group of Asn705 side-chain for apo-1a28 normal MD simulation and holo-1a28 simulation. (B), the distance between Arg788 side-chain amide group and the carboxyl group of Asp709 side-chain for apo-1a28 simulation and holo-1a28 simulation. Black line and dashed black line are used for apo-1a28 and holo-1a28 simulation systems respectively. (PDF) [file pone.0165824.s001.pdf]

**A**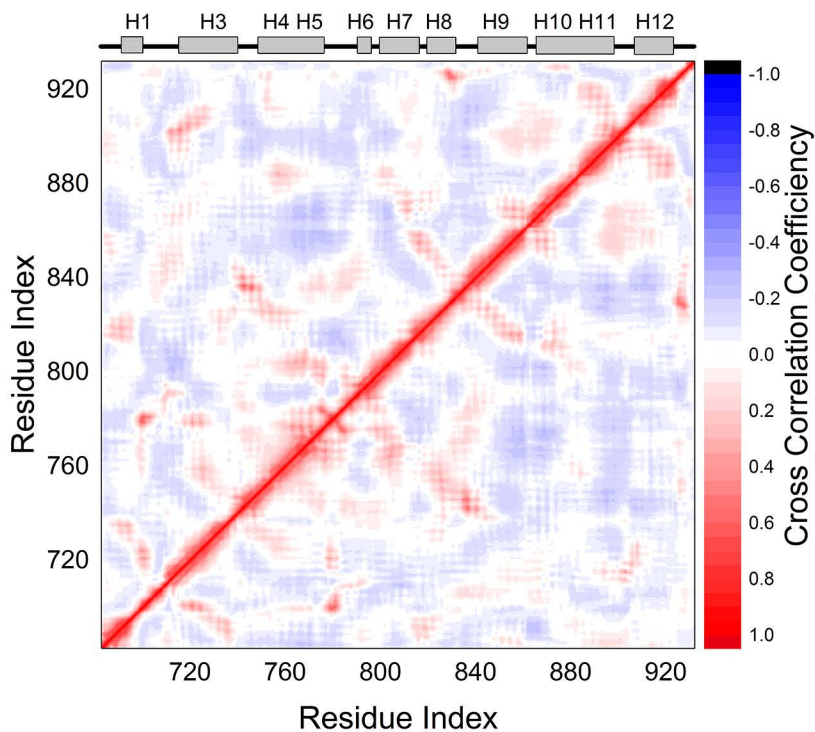**B**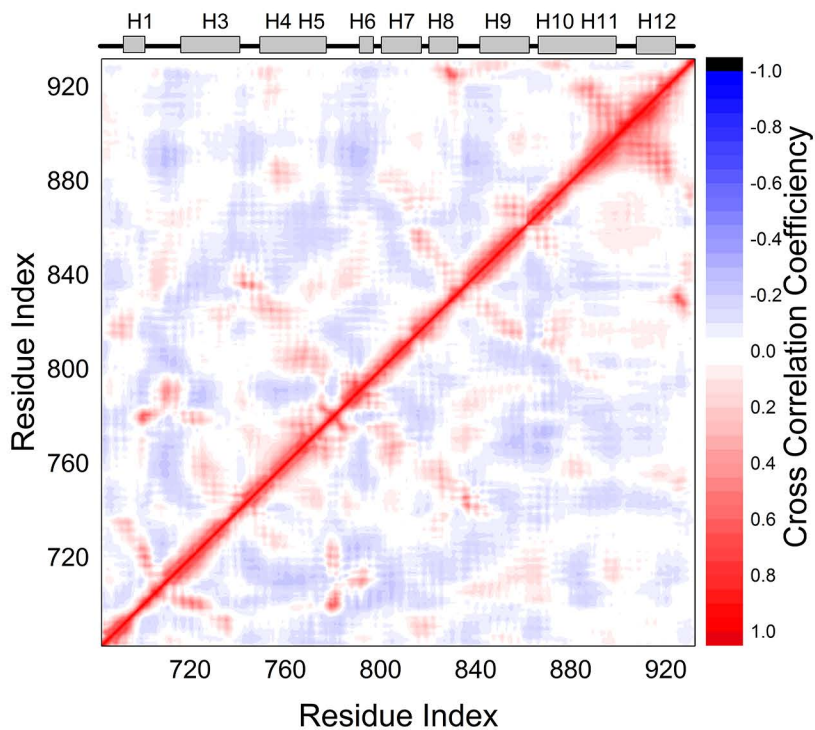

Supplement: S2 Fig — The ccc maps of in apo-form (lower triangle) and holo-form (upper triangle) in agonistic (A) and antagonistic (B) normal MD simulations. The correlation matrices were constructed based on the method of normal MD simulation analysis. The secondary structures are displayed at top of the panels, where “H” represents an α-helix. The color scale is spanning from blue (ccc = -1, fully anti-correlation) to red (ccc = 1, fully correlation), whereas white (ccc = 0) stands for no correlation. (PDF) [file pone.0165824.s002.pdf]

**A**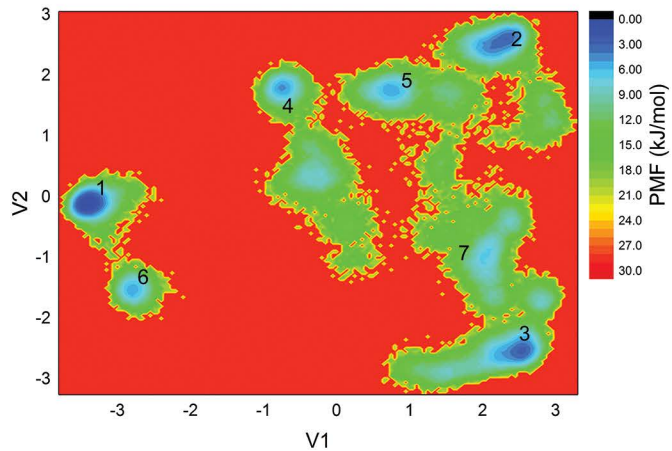**B**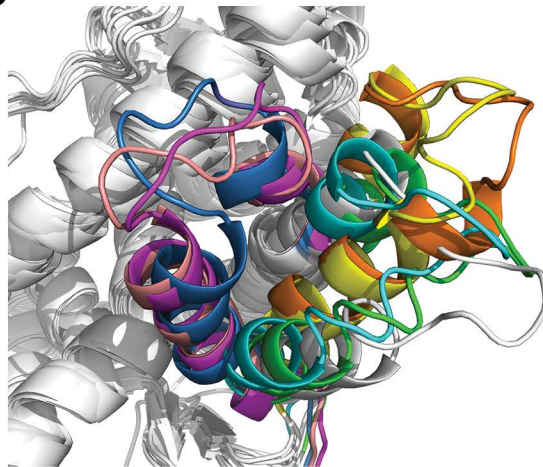**C**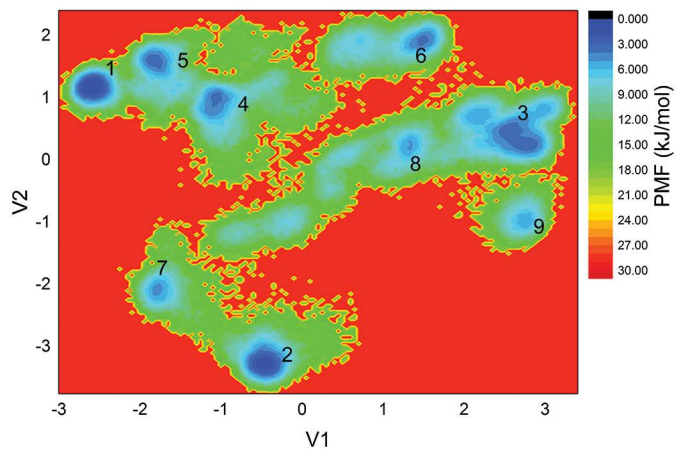**D**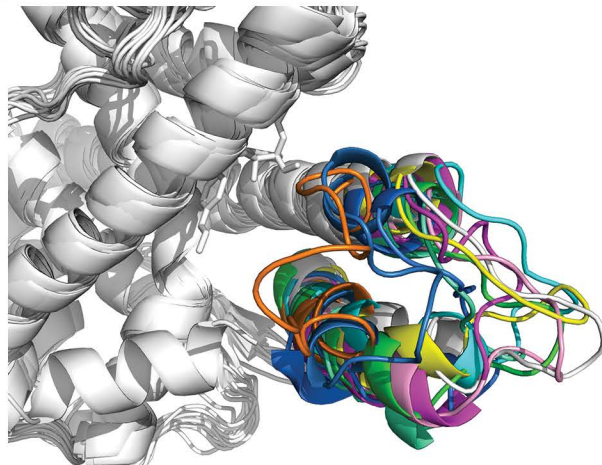

Supplement: S3 Fig — (A), the PMF of the dPCA of the helix 11-loop-helix region in apo-2ovh simulations, where 7 local minima are identified and labeled. (B), the representative structures (labeled as aL1 to aL7) of each minimum in panel A. (C), the PMF of the dPCA of the helix 11-loop-helix 12 region in holo-2ovh simulations, where 9 major local minima are identified and labeled. (D), the representative structures (labeled as hL1 to hL9) of the 9 minima in panel C. From local minima 1 to 7 (panel B), or 1 to 9 (panel D), the representative structures are colored as green, sky blue, yellow, pink, magenta, cyan, orange, green cyan, blue, while the “Ref” labeled structure (gray color) is the crystal antagonistic conformation (PDB ID 2OVH) for reference. (PDF) [file pone.0165824.s003.pdf]

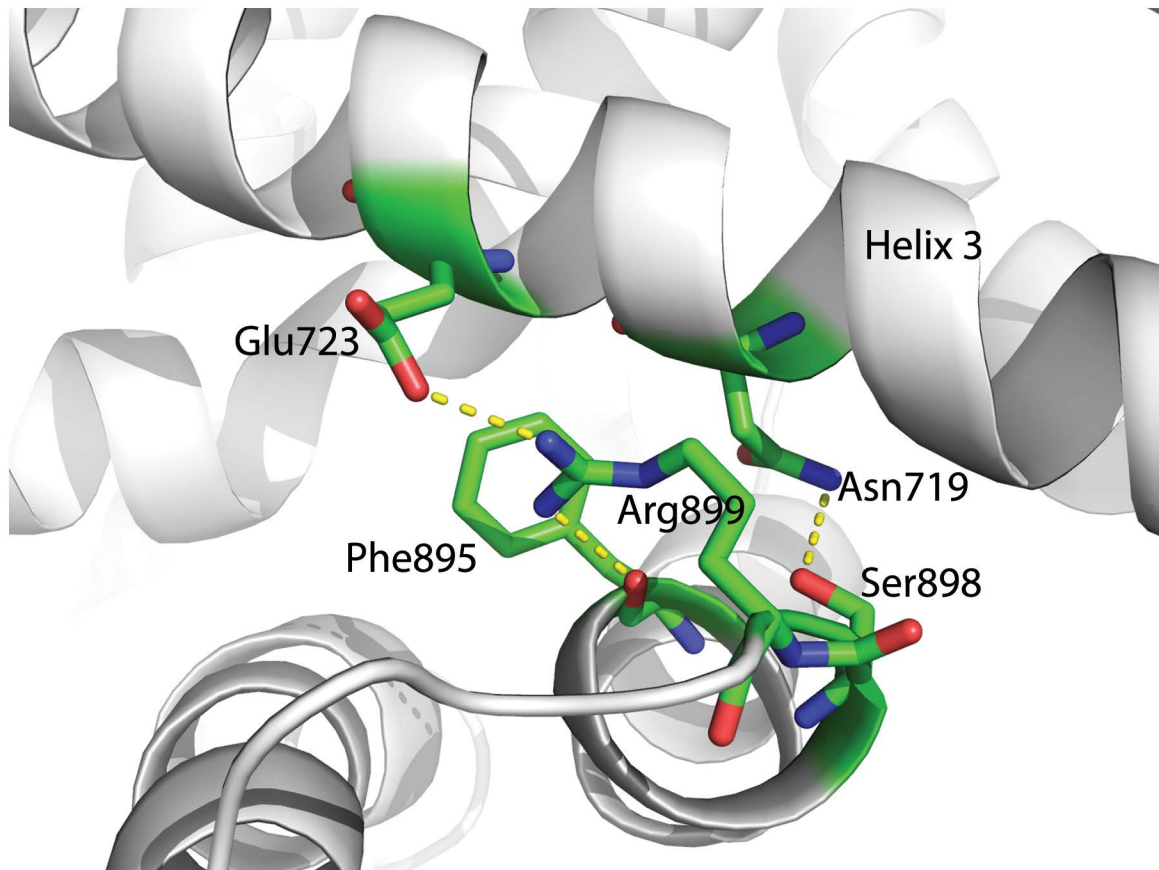

Supplement: S4 Fig — Carbon atoms, nitrogen atoms and oxygen atoms are shown as green, blue and red respectively. Yellow dashed lines indicate close contacts between atoms. (PDF) [file pone.0165824.s004.pdf]

A

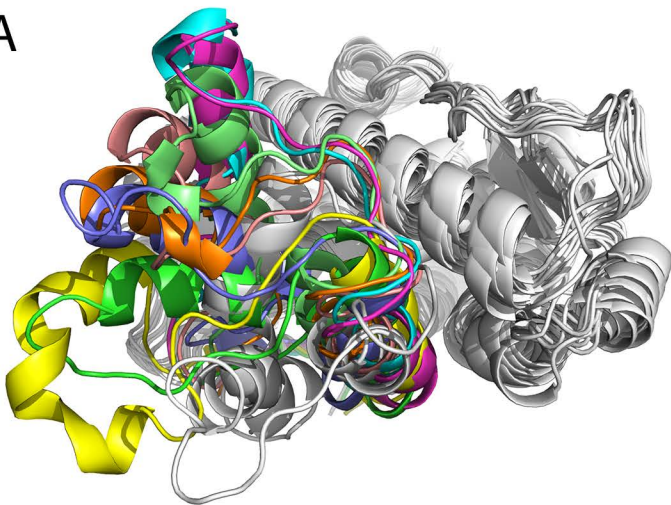

B

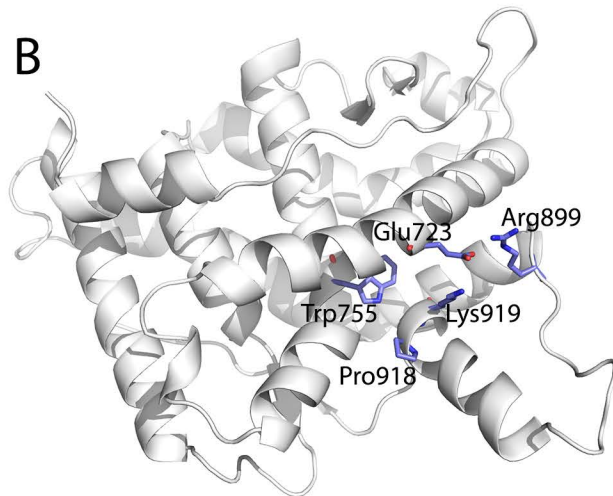

C

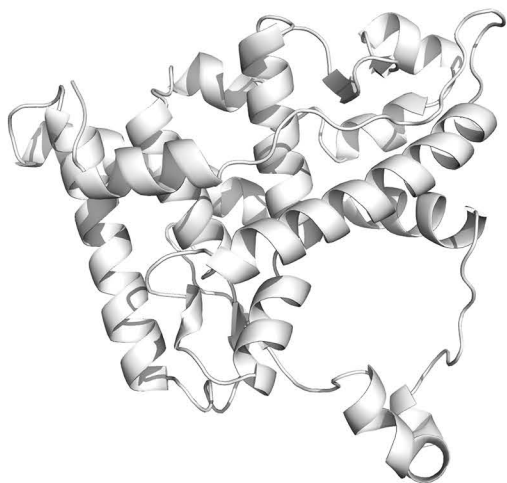

D

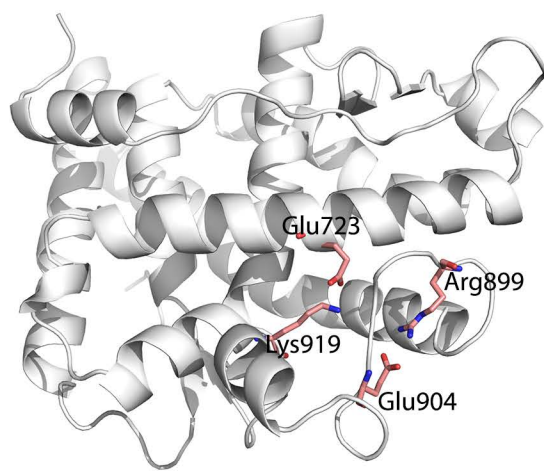

E

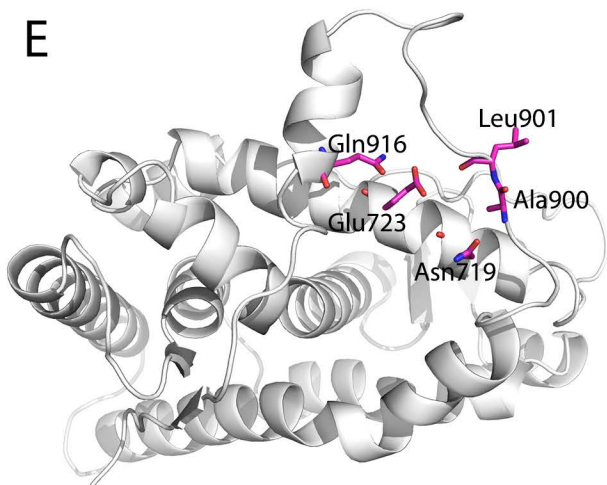

F

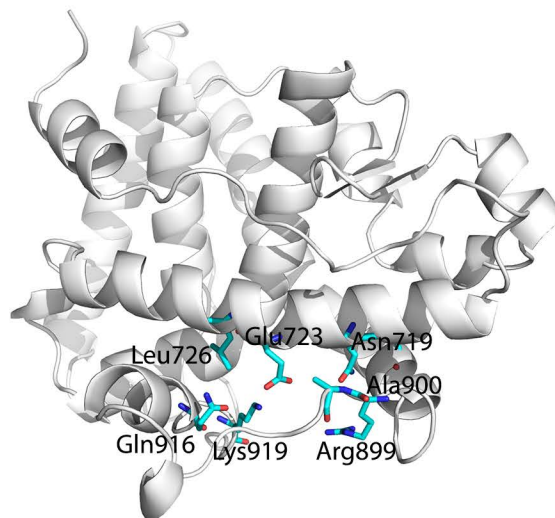

G

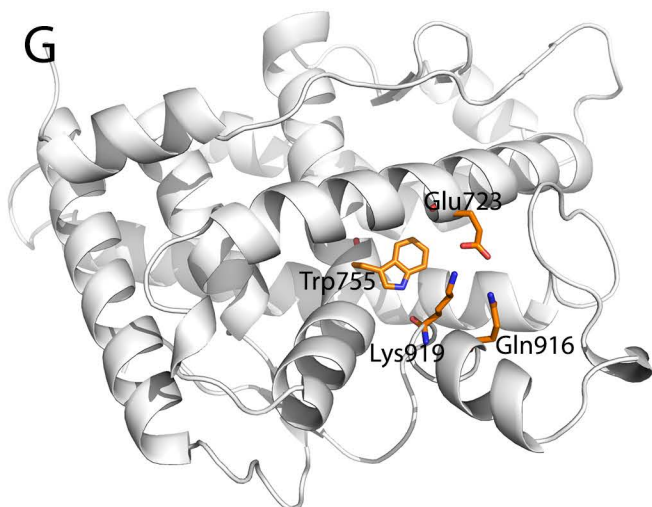

H

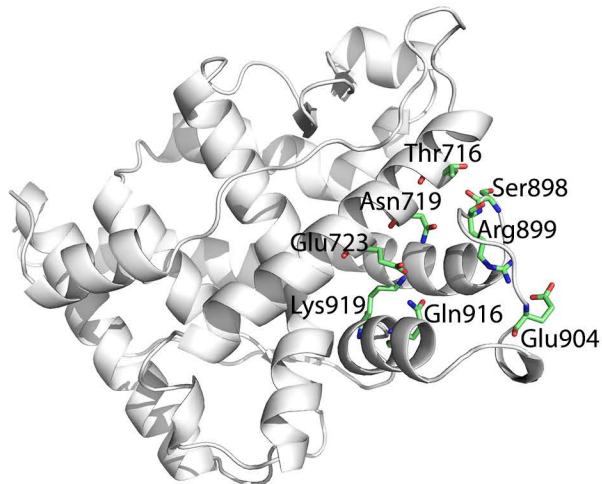

Supplement: S5 Fig — (A), superimposed representative structures. (B-H) detail interactions in flexible helix-loop-helix segment of representative structures from mL2 to mL8. Only the region (residues 892–922) is colored; other parts are shown in gray. Important residues in structures mL2 to mL8, shown as sticks, are colored as green, sky blue, yellow, pink, magenta, cyan, orange, and green cyan respectively. (PDF) [file pone.0165824.s005.pdf]

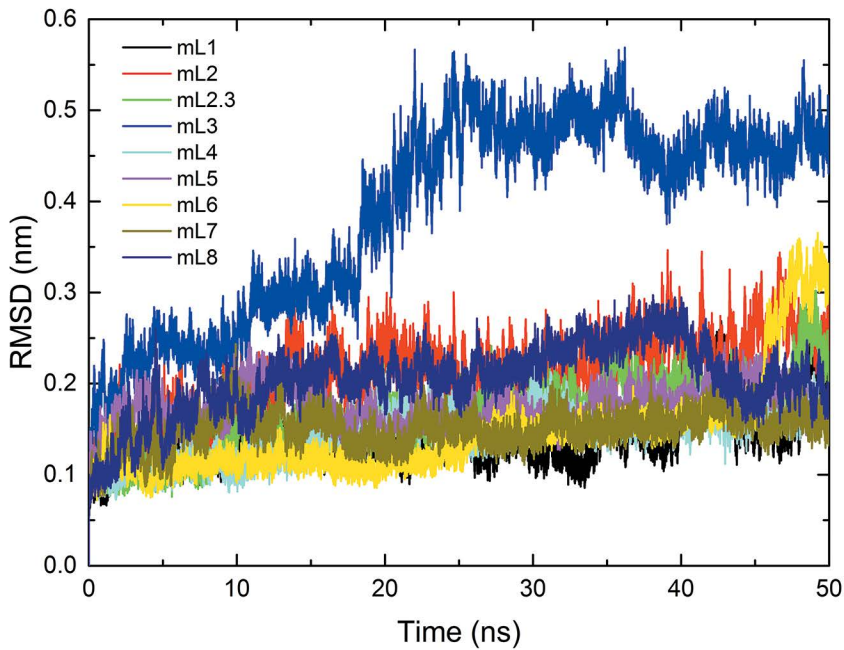

Supplement: S6 Fig — During the 50 ns normal MD simulations, most of the representative structures stay stable (with αC atoms RMSDs are around or within 0.3 nm), while structure mL3 has a rather large RMSD fluctuations which indicates that this mL3 structure is not a real stable states. (PDF) [file pone.0165824.s006.pdf]

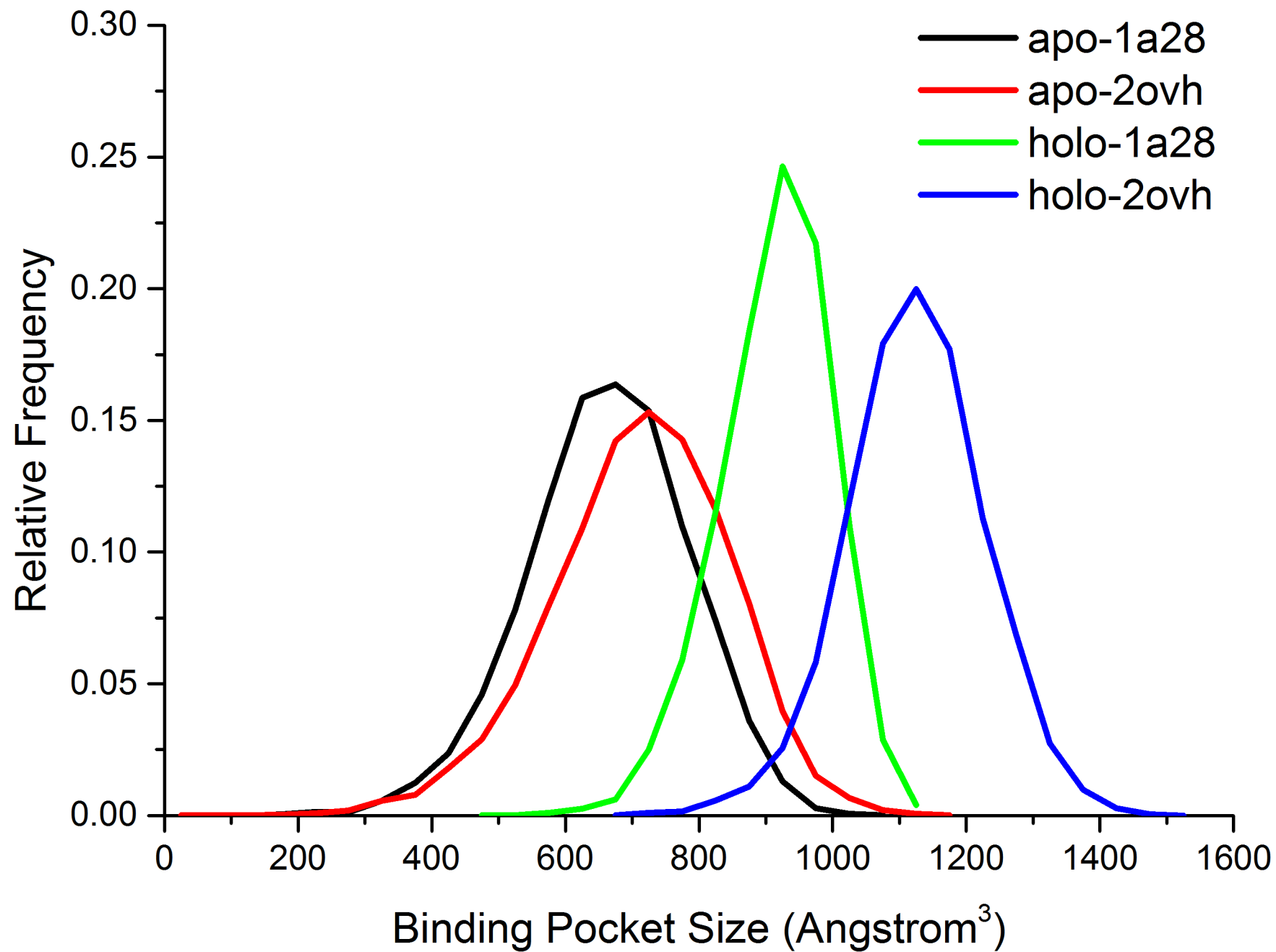

Supplement: S7 Fig — The distance is defined between the COM of three nitrogen atoms in Arg845 side-chain and the COM of aromatic ring in Phe930. Black, red, green and blue lines represent the relative frequency of the distance in apo-1a28, apo-2ovh, holo-1a28 and holo-2ovh normal MD simulations respectively. (PDF) [file pone.0165824.s007.pdf]

**A**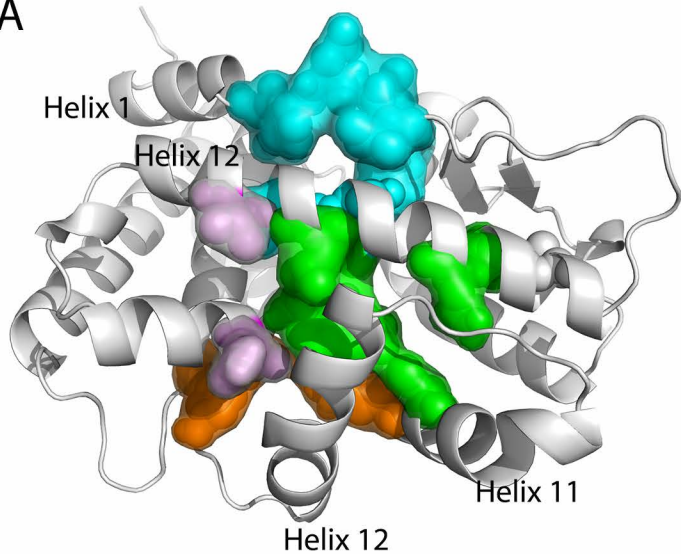**B**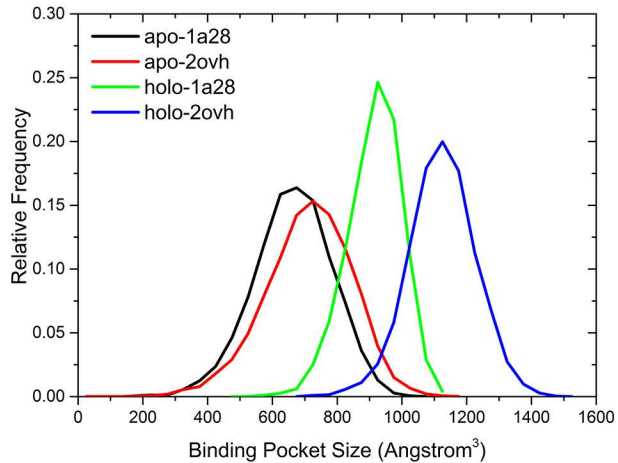

Supplement: S8 Fig — (A), the four druggable sites detected based on the representative conformations from normal MD, umbrella sampling, and metadynamics and plotted on crystal agonistic conformation; their outside residues are shown in different color, whereas the major binding pocket is surrounded green residues. And the other three druggable sites are formed by cyan, orange and magenta residues, respectively. (B) binding pocket size relative frequency distributions in normal MD simulations; black, red, green and blue lines represent the relative frequency of the binding pocket size in apo-1a28, apo-2ovh, holo-1a28 and holo-2ovh normal MD simulations respectively. (PDF) [file pone.0165824.s008.pdf]

**A**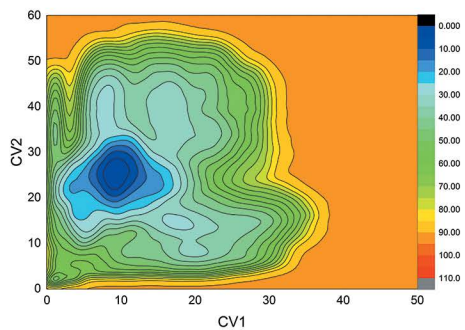**B**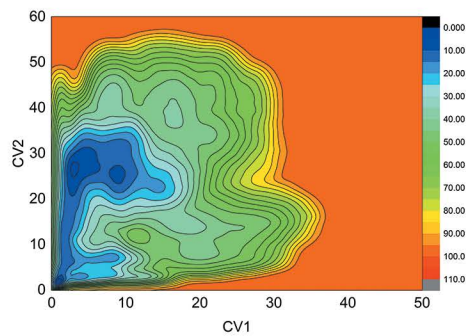**C**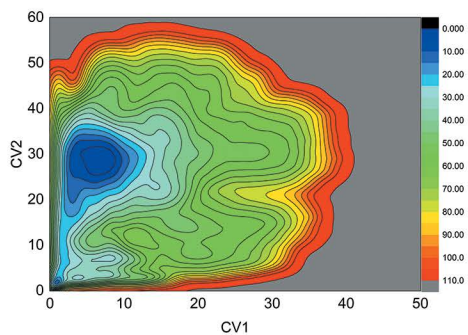**D**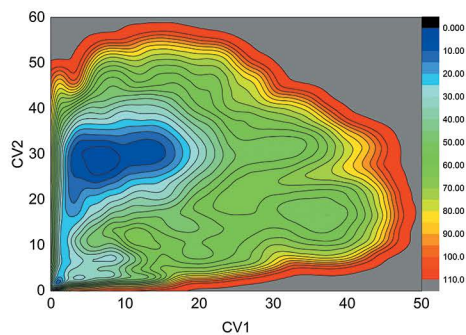**E**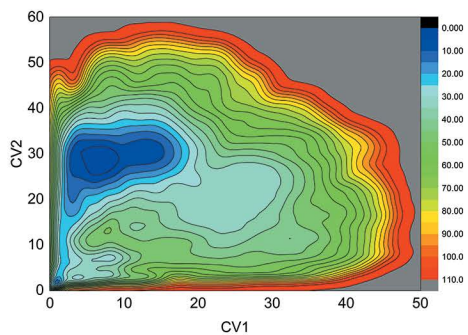**F**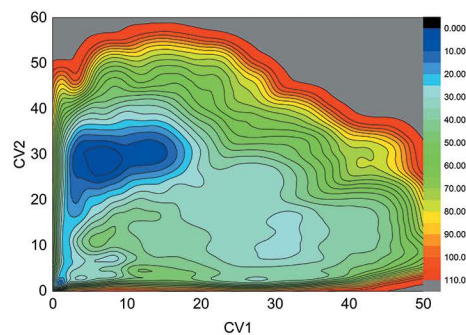**G**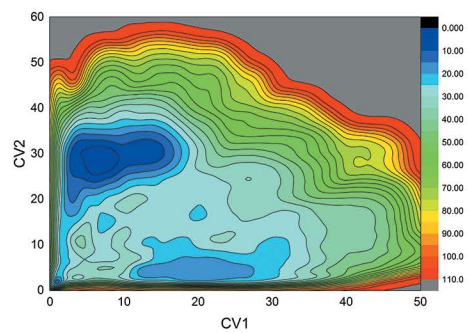**H**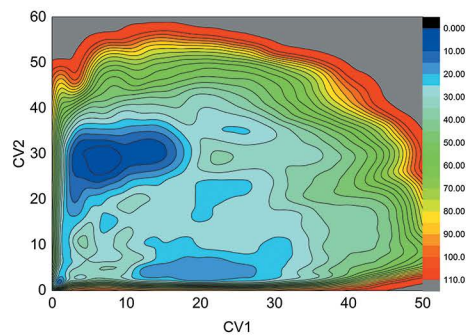

Supplement: S9 Fig — The FESs are constructed based on the Gaussians added from start of the simulation to a specific time point, such as 200 ns (A), 300 ns (B), 400 ns (C), 500 ns (D), 600 ns (E), 700 ns (F), 800 ns (G) and 900 ns (H). The color scales given at the right side of the figures indicate the free energy levels in unit of kJ/mol, while the iso-lines are drawn every 10 kJ/mol. The FESs for 0~700 ns (F), 0~800 ns (G) and 0~900 ns (H) are quite similar from a globular view, thus they could be an indication for the convergence of the metadynamics simulation. (PDF) [file pone.0165824.s009.pdf]

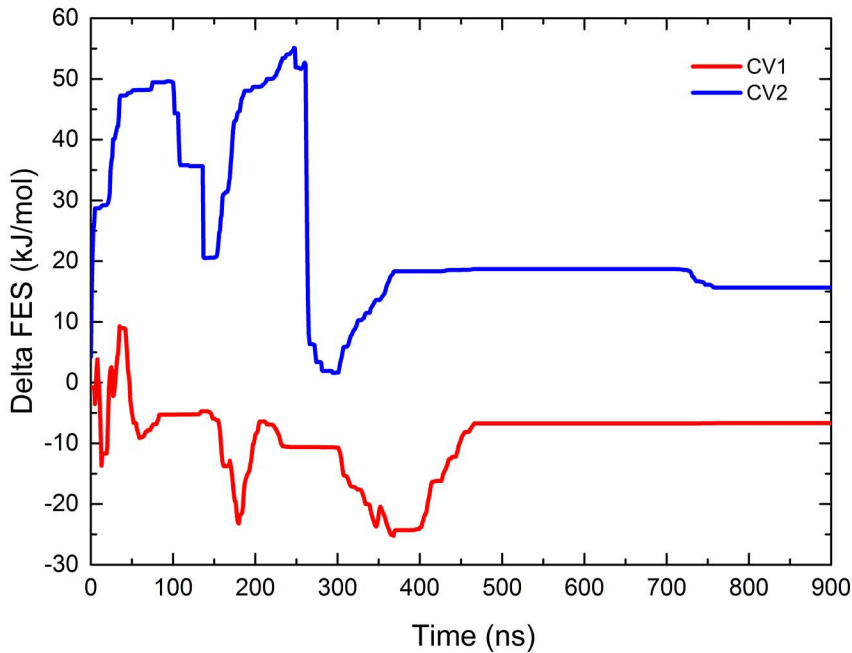

Supplement: S10 Fig — The delta FES changes of NC1 (CV1) and NC2 (CV2) during the whole simulation are presented by the red line and the blue line, respectively. Towards the large part (200 ns) of the simulation, the delta FES tends to be stabilized within a rather small range (1 kJ/mol), therefore it indicates the convergence of the metadynamics simulation. (PDF) [file pone.0165824.s010.pdf]
